# Ptr/CTL0175 Is Required for the Efficient Recovery of *Chlamydia trachomatis* From Stress Induced by Gamma-Interferon

**DOI:** 10.3389/fmicb.2019.00756

**Published:** 2019-04-10

**Authors:** María E. Panzetta, Agustín L. Luján, Robert J. Bastidas, María T. Damiani, Raphael H. Valdivia, Héctor A. Saka

**Affiliations:** ^1^Centro de Investigaciones en Bioquímica Clínica e Inmunología, CONICET, Córdoba, Argentina; ^2^Departamento de Bioquímica Clínica, Facultad de Ciencias Químicas, Universidad Nacional de Córdoba, Córdoba, Argentina; ^3^Instituto de Medicina y Biología Experimental de Cuyo, CONICET, Mendoza, Argentina; ^4^Área de Química Biológica, Facultad de Ciencias Médicas, Universidad Nacional de Cuyo, Mendoza, Argentina; ^5^Center for Host-Microbe Interactions, Department of Molecular Genetics and Microbiology, Duke University, Durham, NC, United States

**Keywords:** *Chlamydia trachomatis*, persistence, penicillin, PTR, IFNγ-induced stress, IFNγ

## Abstract

*Chlamydia trachomatis* is the most common sexually transmitted bacterial pathogen in humans and a frequent cause of asymptomatic, persistent infections leading to serious complications, particularly in young women. *Chlamydia* displays a unique obligate intracellular lifestyle involving the infectious elementary body and the replicative reticulate body. In the presence of stressors such as gamma-interferon (IFNγ) or beta-lactam antibiotics, *C. trachomatis* undergoes an interruption in its replication cycle and enters a viable but non-cultivable state. Upon removal of the stressors, surviving *C. trachomatis* resume cell division and developmental transitions. In this report, we describe a genetic screen to identify *C. trachomatis* mutants with defects in recovery from IFNγ- and/or penicillin-induced stress and characterized a chemically derived *C. trachomatis* mutant strain that exhibited a significant decrease in recovery from IFNγ- but not penicillin-induced stress. Through lateral gene transfer and targeted insertional gene inactivation we identified *ptr*, encoding a predicted protease, as a gene required for recovery from IFNγ-induced stress. A *C. trachomatis* LGV-L2 *ptr-*null strain displayed reduced generation of infectious progeny and impaired genome replication upon removal of IFNγ. This defect was restored by introducing a wild type copy of *ptr* on a plasmid, indicating that Ptr is required for a rapid growth upon removal of IFNγ. Ptr was expressed throughout the developmental cycle and localized to the inclusion lumen. Overall, our findings indicate that the putative secreted protease Ptr is required for *C. trachomatis* to specifically recover from IFNγ- but not penicillin-induced stress.

## Introduction

*Chlamydia trachomatis* is an obligate intracellular bacterium causing a significant burden on human health due to widespread oculo-genital infections. *C. trachomatis* is the leading cause of bacterial sexually transmitted infections (Newman et al., 2015). It is estimated that >70% of *C. trachomatis* endocervical infections are asymptomatic and can persist for prolonged periods of time in the absence of treatment (Stamm, 1999). This contributes to the perpetuity of the transmission cycle, and to the establishment of chronic inflammation and scarring that ultimately lead to complications such as pelvic inflammatory disease, ectopic pregnancy and infertility (Stamm, 1999; Dean et al., 2000; Joyner et al., 2002; Geisler et al., 2008; Haggerty et al., 2010). *C. trachomatis* is also the etiologic agent of trachoma, a long-term ocular infection and the most frequent cause of infectious blindness (World Health Organization [WHO], 2015; Zambrano et al., 2016). Other infections caused by *C. trachomatis* include ophthalmia neonatorum and pneumonia in newborns, inclusion conjunctivitis and less frequently lymphogranuloma venereum (Stenberg and Mardh, 1991; Darville, 2005; White, 2009; Hammerschlag, 2011).

*Chlamydia trachomatis* alternates between developmental forms showing distinct morphological and functional features (reviewed in Hackstadt, 1999; Abdelrahman and Belland, 2005; Tan, 2012; Elwell et al., 2016). The elementary body (EB) is infectious, environmentally stable, smaller, condensed and non-replicative. The reticulate body (RB) is non-infectious, labile in the extracellular milieu, larger and replicative. The infectious cycle begins when EBs attach to epithelial cells and are internalized into a membrane-bound vacuole, termed an inclusion, which serves as a replicative niche for this bacterium. Soon after internalization, EBs transition into RBs, which actively replicate. At the mid-cycle of infection, RBs start differentiating back into EBs in an asynchronous manner, such that at late stages post-infection the inclusion contains numerous EBs. At the end of the cycle, the inclusion occupies most of the host cell cytoplasm and the bacteria are released either by cell lysis or extrusion of intact inclusions (Hybiske and Stephens, 2007). Once in the extracellular environment, EBs are able to infect other cells and continue their infectious cycle. When *C. trachomatis* is stressed during infection, like upon exposure to certain antibiotics, cytokines or nutrient deprivation, these bacteria are able to enter into a viable but non-cultivable state, commonly referred to as *“Chlamydia* persistence” or “*Chlamydia* stress response.” Under this state, *Chlamydia* halts bacterial division and infectious particle generation and usually displays morphologically aberrant and enlarged RBs (aRBs), which are able to rapidly reactivate and resume propagation upon withdrawal of the stressing conditions (reviewed in Wyrick, 2010; Schoborg, 2011; Tan, 2012). The ability to reactivate from a variety of stresses allows *Chlamydia* to ensure long-lasting survival while facing unfavorable growth conditions *in vitro*. Importantly, morphological and clinical evidence suggest that this type of response also occurs *in vivo*. For instance, *C. muridarum* aRBs have been observed in murine endocervical cells using a female genital tract model of infection (Rank et al., 2011; Phillips Campbell et al., 2012), while *C. trachomatis* aRBs were reported in human endocervix (Lewis et al., 2014). In addition, post-gonococcal urethritis reported in *C. trachomatis* co-infected patients treated with penicillin, a known inducer of *Chlamydia* persistence *in vitro* and *in vivo*, may be considered clinical evidence of reactivation upon completion of the antibiotic regime (Wyrick, 2010; Phillips Campbell et al., 2012; Augenbraun, 2015). A variety of stressors have been described to elicit a reversible interruption in *Chlamydia* growth *in vitro*, such us penicillin, IFNγ, deprivation of amino acids, iron, or glucose, as well as exposure to cigarette smoke components, adenosine, co-infection with Porcine Epidemic Diarrhea or Herpes Simplex viruses, infection with a phage and heat-shock (Beatty et al., 1994b; Hsia et al., 2000; Wiedeman et al., 2005; Huston et al., 2008; Pettengill et al., 2009; Vanover et al., 2010; Schoborg, 2011; Prusty et al., 2012; Schoborg and Borel, 2014). Among these, the most extensively studied are IFNγ and penicillin. IFNγ, produced by T-lymphocytes and NK cells, is an important cytokine with critical roles in the immune response against intracellular pathogens (McClarty et al., 2007; Billiau and Matthys, 2009; Aiyar et al., 2014). Different studies confirmed that IFNγ is able to restrict *C. trachomatis* growth in cell culture models of infection (Beatty et al., 1993; Morrison, 2000). In human epithelial cells, this inhibitory effect is mainly due to IFNγ-mediated activation of indoleamine-2,3-dioxygenase (IDO), an enzyme that catabolizes tryptophan into kynurenine, leading to nutritional stress due to *Chlamydia* being tryptophan auxotrophs (Rapoza et al., 1991; Beatty et al., 1993, 1994a). This is in line with observations that IFNγ-responsive IDO-deficient cells or addition of exogenous tryptophan to the culture media, both rescue IFNγ-induced growth restriction in *C. trachomatis* (Beatty et al., 1994a). Interestingly, as opposed to trachoma-associated serovars, *C. trachomatis* genital serovars encode a tryptophan synthase (TrpB) that can synthesize tryptophan from indole (Fehlner-Gardiner et al., 2002). This is in agreement with reports showing that loss-of-function *trpB* mutations are found in ocular but not genital serovars, and are consistent with the observation that addition of exogenous indole allows genital but not trachoma serovars to exit from IFNγ-induced stress (Caldwell et al., 2003; Aiyar et al., 2014). Moreover, a knock-out of *trpB* is sufficient to block recovery after addition of indole in *C. trachomatis* serovar D (Kari et al., 2011). Given that the genital tract microbiota contains indole-producing bacteria, it has been proposed that *C. trachomatis* genital serovars may rely on their tryptophan synthase to evade the antimicrobial effects triggered by IFNγ during *in vivo* infections (Caldwell et al., 2003). In human cells, it has been reported that IFNγ also mediates IDO-independent anti-*Chlamydia* effects via the induction of a family of cell-autonomous p65 guanylate binding proteins (GBPs) (Tietzel et al., 2009; Al-Zeer et al., 2013), although this growth restriction is not always observed (Johnston et al., 2016). Penicillins are beta-lactam antibiotics that inactivate penicillin binding proteins (PBPs), leading to inhibition of peptidoglycan cross-linking, which is required for bacterial cell wall synthesis (reviewed in Sauvage et al., 2008). Addition of penicillin to the culture media was one of the first stimuli shown to induce *Chlamydia* persistence *in vitro* (Beatty et al., 1994b and references therein). Penicillin fails to block EB to RB transition but prevents RB division, rendering inclusions that contain a few aRBs and halting differentiation into EBs until removal of the antibiotic (Galasso and Manire, 1961; Matsumoto and Manire, 1970; Skilton et al., 2009). Even though it is not clear how penicillins induce a stress response in *Chlamydia*, the mechanism is likely linked to the disruption of cell wall synthesis. The observation that amoxicillin, a widely prescribed antibiotic belonging to the penicillin group, induces *Chlamydia* persistence in a murine model of infection (Phillips Campbell et al., 2012), highlights the potential relevance of penicillin-induced persistence *in vivo*.

Several studies have shown that the transcriptional responses triggered in *Chlamydia* are not the same across different stress models, suggesting that specific *Chlamydia* genes participate in regulating the response to different stressful stimuli (Mathews et al., 2001; Belland et al., 2003; Gerard et al., 2004; Goellner et al., 2006; Ouellette et al., 2006; Maurer et al., 2007; Brinkworth et al., 2018). Due to *Chlamydia* being refractory to genetic manipulation until recently (reviewed in McClure et al., 2017; Brothwell et al., 2018; Rahnama and Fields, 2018), the genes involved in such response remain largely unknown. Notably, Muramatsu et al. (2016) carried out a genetic screen aimed at identifying *C. trachomatis* mutants defective for recovery from IFNγ-induced persistence. Using a GFP-expressing *C. trachomatis* lymphogranuloma venereum (LGV) serovar L2 library of mutants, these authors identified six strains displaying impaired recovery upon removal of IFNγ and mapped the mutations responsible for the phenotype to non-synonymous mutations in TrpB (beta subunit of tryptophan synthase), CTL0225 (putative integral membrane protein) and CTL0694 (putative oxidoreductase).

In this study, we carried out a screen to identify *C. trachomatis* genes important for recovery from IFNγ- and/or penicillin-induced stress. We found that *ptr*, encoding a poorly characterized secreted protease, is required for rapid recovery from IFNγ- but not penicillin-induced stress.

## Materials and Methods

### Cell Culture, Infection and *C. trachomatis* Strains

HeLa (CCL-2, ATCC) and Vero cells (CCL-81, ATCC) were cultured in high glucose Dulbecco’s modified Eagle’s medium (Gibco) supplemented with 10% FBS (Natocor) without antibiotics in 5% CO_2_ at 37°C in a humidified atmosphere. Cells were routinely checked for *Mycoplasma* contamination using PCR, as described previously (van Kuppeveld et al., 1992). Infections were done by adding a previously titered suspension of purified EBs at the specified multiplicity of infection (MOI) in culture media to cell monolayers, followed by centrifugation at 2,500 ×*g*, 10°C, 30 min. Then infected cells were transferred to a tissue-culture incubator for the indicated hours post infection (hpi). EB purifications were carried out as described in Saka et al. (2011). EB stocks were titered by assessing the number of Inclusion Forming Units (IFUs) per microliter as described in Nguyen and Valdivia (2012), with minor modifications. Briefly, serial dilutions of EBs were added to Vero or HeLa cell monolayers seeded onto 96-well plates. At 24–30 hpi, cells were fixed with ice-cold methanol (15 min) and stained with rabbit anti-Slc1 (CT043) antibodies (Saka et al., 2011). Inclusions were visualized and counted using a Leica DMI8 epifluorescence microscope (Leica Microsystems). *C. trachomatis* LGV-L2 434/Bu ATCC VR-902B (L2 wt), its rifampin-resistant variant RifR (RifR wt) (Nguyen and Valdivia, 2012) and derivatives used in this study were propagated in Vero or HeLa cells and stored in sucrose phosphate glutamate buffer (SPG) at -80°C. The mutant collection used in this study was obtained using an ethyl methyl sulfonate (EMS)-based mutagenesis method as previously described (Kari et al., 2011; Nguyen and Valdivia, 2012). A detailed description of the collection can be found in a previous publication (Kokes et al., 2015). M275 and the other mutants from the collection were sequenced as specified earlier (Snavely et al., 2014). *C. trachomatis* recombinants were generated by lateral gene transfer and genotyped by TILLING (Till et al., 2006; Kari et al., 2011; Nguyen and Valdivia, 2012). *C. trachomatis* LGV-L2 *ptr::GII* (L2 *ptr::GII*) was generated by insertion of a spectinomycin resistance cassette (*aadA*) in *ptr* (see sections below). L2 *ptr::GII* was complemented with a derivative of pBOMB4R-MCI (GenBank accession no. KF790909) (Bauler and Hackstadt, 2014) constitutively expressing Ptr-FLAG (L2 *ptr::GII* PTR-F). *C. trachomatis* LGV-L2 control strain was also transformed with a derivative of pBOMB4R-MCI expressing Ptr-FLAG (L2 PTR-F). *C. trachomatis* LGV-L2 expressing mCherry was obtained by transformation with the plasmid p2TK2-SW2-IncDPromoter-mCherry (Agaisse and Derre, 2013). *C. trachomatis* LGV-L2 was transformed with pMC71 (pCT143-2HA) (da Cunha et al., 2017) to allow expression of CT143 fused to an HA epitope tag. Vector construction and *Chlamydia* transformation are detailed below. *C. trachomatis* L2 wt and its RifR derivative were used as control strains, as indicated.

### Forward Genetic Screen for Mutants Exhibiting Impaired Recovery Upon IFNγ or Penicillin-Induced Stress

Four identical arrays of the mutant strains were prepared to determine the (i) input (30 hpi), (ii) generation of IFUs in the untreated (control) condition (30 hpi), (iii*)* generation of IFUs post-IFNγ-induced stress (24 h recovery, 48 hpi) and (iv) generation of IFUs post penicillin-induced stress (24 h recovery, 48 hpi). For IFNγ-induced stress and recovery, HeLa cells were seeded in 96-well plates and pre-treated with IFNγ (15 ng/mL, Peprotech) for 24 h, after which cells were washed once with phosphate buffer saline (PBS) and infected with 5 μL of crude cell lysates derived from each mutant in the library in a final volume of 100 μL of DMEM, 10% FBS, IFNγ (15 ng/mL) per well. At the time of infection, cell confluency was ∼80%. At 24 hpi, cells were washed once with PBS, replenished with DMEM, 10% FBS, tryptophan (100 mg/L, Sigma-Aldrich) and let to recover for additional 24 h. At 48 hpi (24 h after IFNγ removal and tryptophan supplementation), cells were lysed with water to release infectious particles and the appropriate volume of SPG 5x was added to obtain a final resuspension of the lysates in SPG 1x. Later, these lysates were used to perform IFU assays and determine infectious progeny generation normalized per input. For penicillin-induced stress and recovery, a similar procedure was carried out except that penicillin G (1 IU/mL, Sigma–Aldrich) was added to HeLa cells at the time of infection and at 24 hpi cells were washed once with PBS, replenished with DMEM, 10% FBS and let to recover for additional 24 h in order to evaluate the generation of IFUs at 48 hpi (24 h after penicillin removal). As control, multiple replicates of the parental *C. trachomatis* LGV-L2 control strain were included in the arrays at various MOIs (0.5, 2, 4) and used to determine the cut-off values for impaired recovery (set as 10 and 20% of IFU/Input of the parental strain for IFNγ and penicillin, respectively). All mutants exhibiting a ≥ 3 fold reduction in IFU/Input compared to the LGV-L2 control strain in the untreated condition were considered to have a growth defect and excluded from the analysis, since these mutants normally generate reduced IFUs even in the absence of stress induction. Inclusions were stained with rabbit anti-Slc1 antibodies followed by Alexa Fluor 594 anti-Rabbit IgG (Life Technologies) and Hoechst 33258 (Life Technologies) was used to stain host cell and *Chlamydia* DNA. Imaging and counting was carried out with Cellomics HTC Arrayscan (Thermo Fisher Scientific) and images analyzed with Cellomics vHCS: View Software v1.6 (Thermo Fisher Scientific) to calculate the IFU values.

### IFNγ and Penicillin Stress Induction and Reactivation Assays

For IFNγ-induced stress and recovery assays, cells were seeded and pre-treated with IFNγ (15 ng/mL) for 24 h. Then, media was removed, and cells were infected with an MOI of 0.8 for each strain in presence of IFNγ (15 ng/mL). After 24 h media was removed, cells were rinsed once with PBS, fresh media supplemented with an excess of tryptophan (DMEM, 10% FBS, tryptophan 100 mg/L) was added and cells were allowed to recover in the incubator for the specified times. At 48, 56, and 72 hpi (corresponding to 24, 32, and 48 h of recovery, respectively) media was removed, cells were lysed with water and resuspended in SPG 1x. These lysates were used to perform IFU assays and determine the progeny generation. Briefly, HeLa cells were infected with serial dilutions of these lysates for 30 h and then fixed with methanol. For penicillin-induced stress and recovery assays, HeLa cells were infected with an MOI of 0.8 for each strain and penicillin (1 IU/mL) was added at the time of infection. After 24 h, cells were rinsed once with PBS, penicillin-free media (DMEM 10% FBS) was added and cells were allowed to recover in the incubator for various times. At 48, 56, and 72 hpi (corresponding to 24, 32, and 48 h of recovery, respectively) media was removed, cells were lysed with water to release infectious particles and the appropriate volume of SPG 5x was added to obtain a final resuspension of the lysates in SPG 1x. These lysates were used to enumerate the number of IFUs and determine infectious progeny generation as described above. Inclusions were identified with an anti-Slc1 antibody and Alexa Fluor 488 anti-rabbit secondary antibody. Inclusions were imaged with a 20x objective in a Leica DMI8 microscope. The number of inclusions was assessed using an ImageJ macro developed in our laboratory. Non-recovery control was always included, these wells were treated for 48, 56, and 72 hpi with penicillin or IFNγ. At all times, progeny was normalized to the input. To calculate the input, HeLa cells infected with an MOI of 0.8 for each strain were methanol-fixed at 30 hpi and inclusions were stained and quantified as described above.

### *Chlamydia* Transformation

*Chlamydia* transformations were performed as described previously (Wang et al., 2011) with minor modifications. Briefly, 10^8^ infectious forming units were mixed with 10–20 μg of the plasmid of interest in CaCl_2_ buffer (50 mM, pH = 7.4) and incubated for 30 min at room temperature. Then, the mix was added to a confluent monolayer of Vero cells seeded in all wells of a 6-well plate. Twelve hours post infection spectinomycin 200 μg/mL (Sigma-Aldrich) or penicillin 1 IU/mL were added, depending on the selectable marker used. At 40–48 hpi crude lysates of infected cells were prepared by osmotic lysis (passage 0/P0) and immediately used to infect a 6-well plate seeded with fresh Vero cells. At 40–48 hpi the harvesting step was repeated, and the crude lysates (P1) used to infect half of a 12-well plate seeded with Vero cells. Passages were repeated every 40–48 h until the appearance of transformants (P2-Px), which normally occurred before P6. Antibiotic was added in all passages for selection. After that, in order to isolate clonal populations transformants were plaque purified as previously described (Matsumoto et al., 1998; Nguyen and Valdivia, 2012) with minor modifications. Briefly, confluent monolayers of Vero cells seeded onto a 6-well plate were infected with serial dilutions of the transformants. At 2 hpi post infection cells were overlayed with DMEM/agarose [0.5% LE agarose (INBIO Highway), 1 × DMEM (Gibco), 10% FBS (Natocor), 50 μg/mL gentamicin (Sigma-Aldrich), 1 × non-essential amino acids (STEMCELL), 200 ng/mL cycloheximide, 200 μg/mL spectinomycin (Sigma-Aldrich) or 1 IU/mL penicillin (Sigma-Aldrich)] and incubated at 37°C and 5% CO_2_ for 8–14 days until plaques became visible. Plaques were picked using a pipette tip and used to infect Vero cell monolayers in 96 well plates. After 48 h of growth, infected cells were lysed by hypotonic disruption for expansion.

### Generation of *Chlamydia* Recombinants Strains by Lateral Gene Transfer

Recombinant strains were generated as previously described (Nguyen and Valdivia, 2012). Briefly, confluent Vero cells seeded on a 6-well plate were coinfected with the rifampin resistant mutant M275 and the spectinomycin resistant wild type strain at an MOI of 3 (one set of 3 wells) and 6 (one set of 3 wells), always with a 1:1 ratio mutant:wild type. At 44 hpi, bacteria were harvested by osmotic lysis and resuspended in 400 μL SPG 1x per well. Then, confluent monolayers of Vero cells seeded onto 6-well plates were infected with serial dilutions of the recombinants (100 μL per well, spanning from undiluted up to 10^-5^). At 2 hpi cells were overlayed with DMEM/agarose [0.5% LE agarose (INBIO Highway), 1 × DMEM (Gibco), 10% FBS (Natocor), 50 μg/mL gentamicin (Sigma-Aldrich), 1 × non-essential amino acids (STEMCELL), 200 ng/mL cycloheximide, 200 μg/mL spectinomycin (Sigma-Aldrich) and 200 ng/mL rifampin (Sigma-Aldrich)] and incubated at 37°C and 5% CO_2_ for 8–14 days until plaques became visible. Recombinant progeny was selected from ∼50 plaques that formed in the presence of 200 ng/mL Rif and 200 μg/mL Spc. Plaque-purified recombinants were further expanded in Vero cells and genotyped by TILLING assay as described elsewhere (Till et al., 2006; Kari et al., 2011) in order to monitor the segregation of mutations in *bioF, recC, oppC* and *ptr* genes that were present in the M275 mutant. Except for one isolate which was isogenic to M275, all other recombinants were wild type for *ptr*. Mutations present in rs9 and rs17 were confirmed by Sanger sequencing (data not shown) with the primers indicated in Supplementary Table S1.

### Generation of *ptr* Null Strain

All cloning steps were performed in *Escherichia coli* DH5a. The insertion site was chosen using the TargeTron^R^ target selection algorithm (Sigma) considering high score and closest proximity to the 5′ ATG start codon. Primers used for re-targeting vector pDFTT3aadA as directed by the TargeTron^R^ manual are listed in Supplementary Table S1. The PCR product was cloned into the BsrGI/HindIII site in pDFTT3aadA plasmid (Lowden et al., 2015). The resulting plasmid pME1 was verified by restriction analysis and Sanger sequencing (data not shown). This plasmid was later used to transform *C. trachomatis* LGV-L2 as described above. Disruption of the *ptr* gene was confirmed by PCR and sequencing of the PCR product obtained with primers Ptr2F and Ptr2R (Supplementary Figures S4, S5).

### Complementation of L2 *ptr*::*GII*

To complement the LGV-L2 *ptr* knock-out strain (L2 *ptr::GII*), *ctl0175* was amplified by PCR from *C. trachomatis* LGV-L2 purified genomic DNA (DNeasy kit, Qiagen) using F-rpoBPtr and R-PtrFLAG primers (Supplementary Table S1). The PCR product was cloned into SacII/KpnI-digested pBOMB4R-MCI (Bauler and Hackstadt, 2014) and transformed into *E. coli* DH5a. The resulting plasmid pBOMB-ptrF was verified by restriction analysis and Sanger sequencing (data not shown) and used to transform *C. trachomatis* LGV-L2 and L2 *ptr::GII* strains as described above.

### One-Step Growth Curve

HeLa cell monolayers were infected with the specified *C. trachomatis* strains at an MOI of 0.6 until the indicated time points (6, 20, 24, 30, 42 hpi), at which bacteria were harvested by osmotic lysis. Serial dilutions of these lysates were used to infect HeLa cells. Infected cells were fixed with methanol at 30 hpi, labeled with anti-Slc1 and with a secondary Alexa Fluor 488-conjugated anti-rabbit IgG. Inclusions were imaged at 20x magnification with a Leica DMI8 epifluorescence microscope and counted to determine output IFUs. Output IFUs were normalized by input IFUs (initial inoculum).

### Determination of Inclusion Size

To measure inclusion size in untreated (24 and 30 hpi), IFNγ-treatment (IFN: cells were pre-treated for 24 h with 15 ng/mL of IFNγ, then infected for 24 h in presence of IFNγ), or IFNγ-recovery (IFN-Rec: at 48 hpi, 24 h after removal of IFNγ and addition of tryptophan) conditions, bacteria were labeled with anti-Slc1 antibodies (Saka et al., 2011), and the area of the inclusions was measured and expressed in arbitrary units using an ImageJ macro.

### Genome Copy Number Quantification

For determination of genome copy number, DNA was isolated from infected HeLa cells at the indicated conditions (with IFNγ at 2, 24, 48, and 72 hpi; post IFNγ after Trp addition at 48 and 72 hpi) using the DNeasy kit (Qiagen) and following manufacturer’s instructions. Reactions were prepared with PowerUp SYBR Green Master Mix and quantitative PCR was performed on a 7500 Real-Time PCR device (Applied Biosystems). *ompA* was PCR amplified from *C. trachomatis* LGV-L2 genomic DNA using FompA_BamHI and RompA_SalI (Supplementary Table S1). PCR product was cloned into the BamHI/SalI site of pET24d and transformed into *E. coli* DH10B. pET24d_OmpA purified plasmid was quantified with Qubit (Thermo Fisher Scientific) and used to generate a standard curve. *ompA* copies were quantified using primers MOMP_F and MOMP_R (Supplementary Table S1). Cycling conditions were: 10 min at 95°C, followed by 40 cycles of 95°C for 15 s and 60°C for 1 min.

### Transmission Electron Microscopy

To evaluate the ultrastructural features of HeLa cells infected with the specified strains, the infected cell monolayers were fixed at the indicated hpi with 2.5% glutaraldehyde/0.1M sodium cacodylate and scraped from the wells. Fixed cells were centrifuged 5 min at 1500 rpm and the pellets post-fixed, de-hydrated, embedded, cut, and examined as described previously (De Paul et al., 2009). RifR and M275 samples were processed in Duke Electron Microscopy Facility; L2 wt, L2 *ptr::GII* and L2 *ptr::GII* PTR-F samples were processed by the Electron Microscopy Center of Facultad de Ciencias Médicas, Universidad Nacional de Córdoba. To enumerate the numbers of EBs and RBs, at least 10 inclusions were analyzed per condition. Images were processed with Adobe Photoshop CS6 (Adobe Systems Inc.,).

### Immunodetection Assays

Primary rabbit antibodies used were anti-HA epitope tag (Life Technologies, 715500), anti-Slc1 (CT043) (Saka et al., 2011) and anti-IncA (Cocchiaro et al., 2008). Primary mouse antibodies were: anti-MOMP (Abcam, ab20881) and anti-FLAG M2 (Sigma-Aldrich, F3165). Mouse polyclonal Abs were raised against *C. trachomatis* LGV-L2 Ptr peptide (GenScript) as described below. Secondary antibodies were Alexa Fluor 488 or 594 anti-mouse or anti-rabbit (Jackson Immuno Research). DNA was stained with Hoechst 33258 (Life Technologies). Immunofluorescence images were acquired on a Leica DMI8 epifluorescence microscope and on an Olympus FV1000 confocal microscope. Images were processed with ImageJ 1.51n and/or Adobe Photoshop CS6. For co-localization analysis of Ptr and Ptr-FLAG (Figure 5), BAR plugin for ImageJ was used (data repository: DOI 10.5281/zenodo.28838). For deconvolution of z-stack images (image used for Figure 5E and Supplementary File S1), the Huygens Professional Software (Scientific Volume Imaging, Netherlands) was used, using the classical maximum likelihood estimation algorithm, a theoretical PSF, a signal-to-noise ratio of 10, a maximum of 40 iterations and an automatic background search. The co-localization movie (Supplementary File S1) of Ptr (green) and CT143 (red) was generated with Huygens Surface Renderer software (Scientific Volume Imaging B.V.) with the following transparency settings: green channel 100%, red channel, 0%. For western blots, protein extracts were generated by lysing cells or tissues as recommended by Biorad protocols and heated at 90°C for 5–10 min. Equal amounts of protein were resolved by SDS/PAGE, transferred onto 0.45 μm nitrocellulose membranes (Biorad), and incubated overnight with the corresponding primary antibodies followed by fluorescently labeled secondary antibodies (LI-COR Biosciences). Bands were visualized with Odyssey CLx imaging system (LI-COR Biosciences). To perform fractionation of bacteria (pellet) and HeLa lysates (supernatant), cells were infected with L2 PTR-F or L2 *ptr::GII* PTR-F strains and gently lysed with 400 μL of water (15 min, 37°C) at 30 hpi. Lysates were centrifuged (15,000 rpm, 4°C, 15 min) to separate the bacterial pellet from the supernatant. To lyse bacteria, the pellet was resuspended in lysis buffer [20 mM Tris.HCl pH 8, 5 mM EDTA, 50 mM NaCl, 1% Triton X100, 1 mM PMSF, protease inhibitor cocktail (Roche), 1% SDS, 15 mM DTT,14.3 mM 2-mercapto ethanol]. Both bacterial and host cell lysates were mixed with Laemmli buffer (2x final), sonicated (20% amplitude, 1 min.) on ice, incubated 10 min at 65°C and boiled for 10 min. Protein lysates were resolved by SDS-PAGE and immuno-blotted for FLAG (Ptr-FLAG) and MOMP.

### Polyclonal Antibody Production

Purebred male C57BL/6 mice (aged 10 weeks) housed at the vivarium of the Instituto Mercedes and Martín Ferreyra were used to generate mouse polyclonal antiserum against *C. trachomatis* LGV-L2 Ptr peptide. Mice were maintained in the SPF animal facilities with environmental enrichment that meet the conditions of the Guide to the Care and Use of Experimental Animals (CICUAL N° 001-2017). Immune Epitope Database Analysis Resource^1^ was used for peptide selection. Regions with predicted beta-turn conformation, high antigenicity, surface accessibility, and hydrophilicity were positively considered. The selected peptide (SSPYAAPSYYPQRKP) was synthesized with Keyhole Limpet Hemocyanin (KLH) conjugation on the N-terminal end (GenScript). Cysteine was added at the N-terminus for KLH conjugation. The chosen peptide is located in CTL0175 protein sequence between amino acids 746 and 760. This region is upstream the predicted stop codon in M275 (W801^∗^). Two male C57BL/6 mice were intraperitoneally injected with 200 μg of antigen emulsified with Sigma Adjuvant System (SAS) (Sigma-Aldrich, St. Louis, MO, United States) (1:1) in a final volume of 200 μl on days 1, 21, and 45. Polyclonal antisera were obtained after the second and the third immunization and the production of anti-Ptr antibodies were analyzed by immunofluorescence (IF) and immunoblotting. The anti-Ptr antibody obtained was useful for IF but not for immunoblot assays. In order to determine antibody specificity in IF, anti-Ptr antibodies were incubated with the synthetic Ptr peptide (0.5 mg) overnight at 4°C on a rocker, and then used to label infected HeLa cells with a secondary antibody Alexa Fluor 488.

### Statistics

Statistical analysis was performed using GraphPad Prism 5.0 (^∗^*P* < 0.05; ^∗∗^*P* < 0.01; ^∗∗∗^*P* < 0.001; ns, not significant, *P >* 0.5). Tests performed are detailed in the corresponding figure legends.

## Results

### A Genetic Screen Identifies *C. trachomatis* Mutants Impaired for Recovery From IFNγ- and Penicillin-Induced Stress

To identify *C. trachomatis* genes relevant for recovery from IFNγ- and penicillin-induced stress, we screened a previously described collection of ∼ 900 chemically mutagenized *C. trachomatis* LGV-L2 strains (Kokes et al., 2015). We first set up stress and recovery conditions for *C. trachomatis* LGV-L2 in HeLa cells using IFNγ or penicillin as stressors. For IFNγ-induced stress, HeLa cells were pre-treated for 24 h with 15 ng/mL of IFNγ, then infected for 24 h in presence of the stressor and allowed to recover for 24 h in IFNγ-free media supplemented with tryptophan. For penicillin-induced stress, HeLa cells were infected for 24 h in presence of 1 IU/mL of penicillin and then allowed to recover for 24 h in penicillin-free media. As previously reported (Tamura and Manire, 1968; Beatty et al., 1993), we observed that in continuous presence of the stressors, the generation of infectious progeny was drastically reduced compared to the untreated control. At 24 h upon removal of the stressors, the production of infectious progeny peaked (up to 4 and 3 orders of magnitude in the case of IFNγ and penicillin, respectively) compared to the stressed condition. However, progeny generation post-recovery still was significantly lower (1 and 2 orders of magnitude in the case of IFNγ and penicillin, respectively) compared to the untreated control (Supplementary Figure S1A). In the presence of penicillin, inclusions clearly showed *Chlamydia* forms compatible with the typical enlarged, aRBs (Supplementary Figure S1B). In the presence of IFNγ, inclusions were smaller but the observation of typical aRBs was not as evident as compared to penicillin treatment (Supplementary Figures S1B–D). This is consistent with the observation that *C. trachomatis* LGV-L2 is less sensitive to IFNγ compared to other serovars (Morrison, 2000) and may explain why IFNγ-induced aRB formation were more difficult to observe (reviewed in Bavoil, 2014). In our culture conditions, addition of 15 ng/ml of IFNγ clearly prevented infectious progeny generation but aRB formation was not readily observed. This may indicate that IFNγ-treatment managed to cause nutritional stress due to reduced tryptophan availability, but did not induce a “fully persistent” state. Because higher concentrations of IFNγ were toxic to cell monolayers (data not shown), we did not increase the levels of this cytokine in our screen. After 24 h of recovery from IFNγ-induced stress, inclusions were morphologically indistinguishable from the untreated control. In contrast, removal of penicillin did not completely revert the abnormal RB morphology (Supplementary Figure S1B) as has been previously reported (Skilton et al., 2009).

**FIGURE 1:**
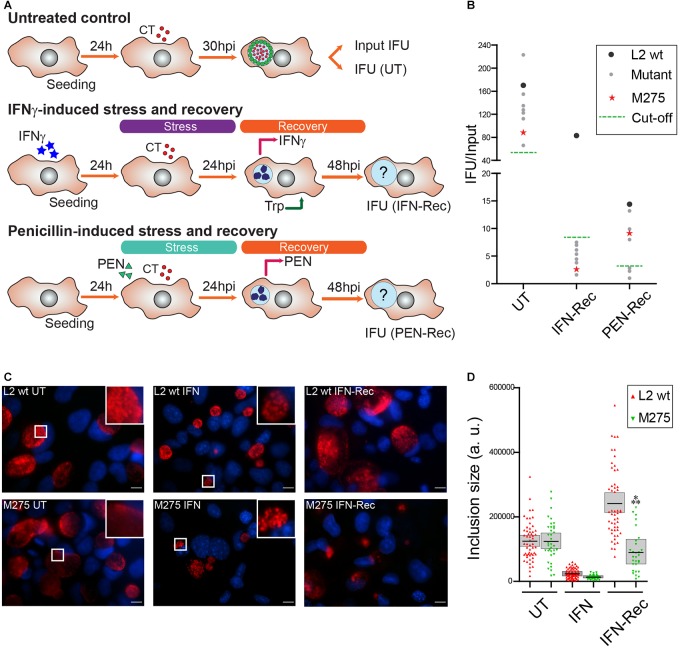
A genetic screen identifies a *C. trachomatis* mutant impaired for recovery from IFNγ-induced stress. **(A)** Schematic representation of the phenotypic screen used to identify *C. trachomatis* mutants impaired for recovery from IFNγ- or penicillin (PEN)-induced stress. Four identical arrays of the mutant strains were prepared in 96 well plates; one array was used to determine the input of infectious forming units (Input IFU) while the other three were used to determine output IFUs (IFU) at the indicated hours post-infection (hpi) in either the untreated (UT, upper panel), IFNγ-recovery (IFN-Rec, middle panel) or PEN-recovery condition (PEN-Rec, lower panel). For IFNγ-induced stress, cells were pre-treated for 24 h with 15 ng/ml of IFNγ prior to infection. At 24 hpi the stressor was removed and tryptophan (100 mg/L) was added to the culture medium to evaluate the recovery of IFUs at 48 hpi (IFN-Rec). For PEN-induced stress, penicillin (1 IU/mL) was added at the time of infection and removed at 24 hpi to evaluate the recovery of IFUs at 48 hpi (PEN-Rec). As control, wild type *C. trachomatis* LGV-L2 was included in the arrays. **(B)** *C. trachomatis* mutants with impaired production of infectious progeny normalized per input (IFU/Input) upon recovery from IFNγ- or PEN-induced stress (gray dots, red star, *n* = 8) compared to wild type *C. trachomatis* LGV-L2 (L2 wt, black dots). The mutant strain M275 (red star) reproducibly showing impaired generation of IFUs in the recovery from IFNγ- but not PEN-induced stress was chosen for further characterization. **(C)** Fluorescence microscopy micrographs of HeLa cells infected with LGV-L2 (L2 wt, upper panels) or M275 (lower panels) in either untreated (UT, 30 hpi), IFNγ-treated (IFN: cells pre-treated for 24 h with 15 ng/mL of IFNγ, then infected for 24 h in presence of IFNγ), or IFNγ-recovery (IFN-Rec: at 48 hpi, 24 h after removal of IFNγ and addition of tryptophan). Insets highlight representative inclusions under UT and IFN conditions. *C. trachomatis* inclusions were labeled with anti-Slc1 (red) and DNA was stained with Hoechst (blue). Scale bar represents 10 μm. **(D)** Comparison of inclusion sizes (expressed in arbitrary units, a. u.) between LGV-L2 (L2 wt) and M275 in UT, IFN or IFN-Rec conditions. Asterisks indicate statistically significant differences (?*P* < 0.001) between M275 and L2 wt as determined by two-way ANOVA and Bonferroni post-test (bar, mean; shaded area, SEM, *n* = 3).

We next reasoned that *C. trachomatis* mutants exhibiting reduced levels of infectious progeny upon removal of the stressors, are defective either in their ability to survive during stress or to resume growth/progeny generation. To identify such mutants, we infected HeLa cells with individual chemically derived mutant strains in the presence of IFNγ or penicillin, then removed the stressors at 24 hpi and allowed the bacteria to recover for an additional 24 h (Figure 1A). We then measured the generation of infectious progeny for each mutant by counting inclusion forming units (IFUs) in untreated condition at 30 hpi (UT), or at 24 h of recovery from IFNγ- or penicillin-induced stress (IFN-Rec and PEN-Rec conditions, respectively). To account for variations in infectivity among mutant strains, output IFUs were normalized to input IFUs (IFU/Input). A defective mutant was defined as that producing ≤10% and/or ≤20% of IFU/Input upon recovery from IFNγ- and penicillin-induced stress, respectively, compared to the LGV-L2 control strain. We identified 8 mutants displaying reduced IFU generation upon recovery from IFNγ-induced stress, 3 of which were also defective for reactivation upon penicillin-induced stress (Figure 1B). We focused our analysis on mutant M275, which reproducibly showed impaired recovery from IFNγ- but not penicillin-induced stress. To determine the effect of IFNγ treatment on inclusion development, we measured inclusion size for wild type and M275 strains. In the absence of stressors, wild type and M275 strains developed inclusions that were similar in size. Interestingly, after removal of IFNγ, M275 showed significantly smaller inclusions compared to the parental strain (Figure 1C,D).

### M275 Is Defective for Recovery From IFNγ- but Not Penicillin-Induced Stress

To determine if the stress-recovery defect of M275 was specific for IFNγ-induced stress, we analyzed the generation of IFUs upon recovery from IFNγ- and penicillin-induced stress at three different time-points: 48, 56, and 72 hpi, corresponding to 24, 32, and 48 h of recovery, respectively. In the UT and PEN-Rec conditions M275 generated similar IFUs/Input compared to the RifR wt parental strain (Supplementary Figures S2A, S2C). In contrast, in the IFN-Rec condition M275 showed a significant reduction in IFU generation in all three time-points analyzed (Supplementary Figure S2B). These results confirm that M275 exhibits a defect specifically in the recovery from IFNγ- but not penicillin-induced stress, indicating that *C. trachomatis* recover from these two stresses via different mechanisms. We next analyzed HeLa cells infected with the control strain or M275 by electron microscopy to evaluate the ultrastructural features of developmental forms in the UT and IFN-Rec conditions. In the UT condition, M275 displayed no apparent anomalies compared to the control strain (Supplementary Figure S2D). However, M275 showed a significant reduction in both the absolute number of EBs per inclusion as well as in EB/RB ratio upon recovery from IFNγ-induced stress (Supplementary Figures S2D,E). To determine if the reduction in IFUs and the EB/RB ratio observed in M275 was associated to a defect in genome replication during recovery from IFNγ-induced stress, we used real-time PCR and assessed the rate of genome accumulation. We found that compared to the parental control strain, M275 exhibited reduced genome accumulation during recovery from IFNγ-induced stress (Supplementary Figure S2F).

### Recombinant Analysis Indicates That Neither Mutations in recC or oppC Are Linked to the Phenotype Observed in M275

Whole-genome sequencing of M275 revealed 6 single nucleotide variants (SNVs) compared to the parental RifR wt strain (Table 1). Two of them (G126A and C204T) lead to synonymous mutations. SNVs in *recC* (C1003T)*, oppC* (G541A) and *bioF* (G566A) are predicted to cause single amino acid substitutions (Table 1). One SNV located in *ptr* (C2403T) is predicted to cause a premature stop codon (W801^∗^) and thus it is the most likely to alter the function of the resulting protein. To determine which mutations are linked to the stress-recovery defect observed for M275, we generated recombinant strains by performing co-infections between the rifampin resistant M275 and a spectinomycin resistant LGV-L2 strain followed by selection of double drug-resistant recombinants by plaque-purification (Supplementary Figure S3A). A total of ∼50 recombinants were genotyped by TILLING assay (Till et al., 2006; Kari et al., 2011) (data not shown). Two recombinant strains, rs9 and rs17, harbored a single SNV in *oppC*, and SNVs in *oppC* and *recC*, respectively, leading to single amino acid substitutions (Table 1). Both recombinants were tested in IFNγ-induced stress and recovery assays and compared to M275 and RifR wt. In the UT condition, generation of IFUs for both recombinants was similar to RifR wt, as expected (Supplementary Figure S3B). Unlike M275, which consistently showed a significant reduction in IFU generation in all three time-points analyzed upon recovery from IFNγ-induced stress, rs9 and rs17 produced higher levels of IFUs, similar to those observed for RifR wt (Supplementary Figure S3C). These results suggested that neither *recC* nor *oppC* contributed to impaired recovery from IFNγ-induced stress.

**Table 1:** Single nucleotide variants present in the genome of M275.

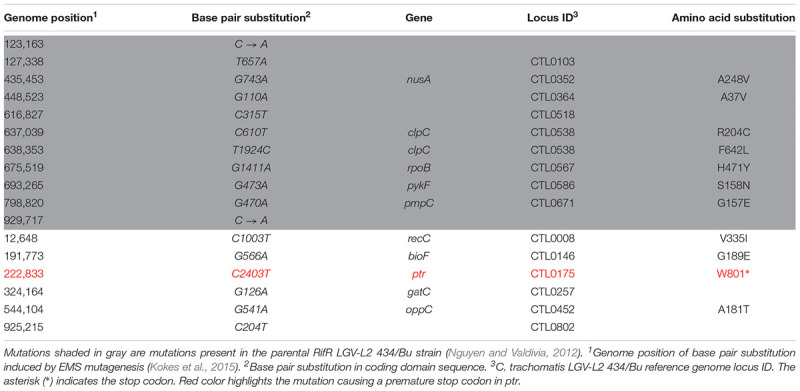

### Impaired Recovery From IFNγ-Induced Stress Is Linked to the Loss of Ptr Expression

We turned our attention to the remaining candidate mutations present in M275: *bioF* and *ptr*. *bioF* is predicted to encode a 7-keto-8-aminopelargonate (KAPA) synthase, which may participate in the first step of synthesis of biotin by converting pimeloyl-CoA to KAPA (Fisher et al., 2012). *ptr* corresponds to locus CTL0175 in *C. trachomatis* LGV-L2 and encodes an uncharacterized putative Zn^2+^-dependent metalloprotease with peptidase domains of the M16 superfamily. Since the SNV present in *ptr* leads to a premature stop codon, we focused on this gene as the most likely candidate responsible for the impaired recovery of M275 from IFNγ-induced stress. To evaluate Ptr expression in M275, we generated a polyclonal antibody against a synthetic peptide corresponding to amino acid positions 746–760 in Ptr protein sequence, which is located 5′ to the predicted nonsense codon. By immunofluorescence microscopy (Supplementary Figure S3D) Ptr labeling was detected in the RifR wt strain, but not in M275. Furthermore, the specificity of the anti-Ptr antibody was confirmed by incubation with an excess of the immunizing Ptr peptide, which led to a marked reduction of the anti-Ptr fluorescence signal (Supplementary Figure S3E).

**FIGURE 2:**
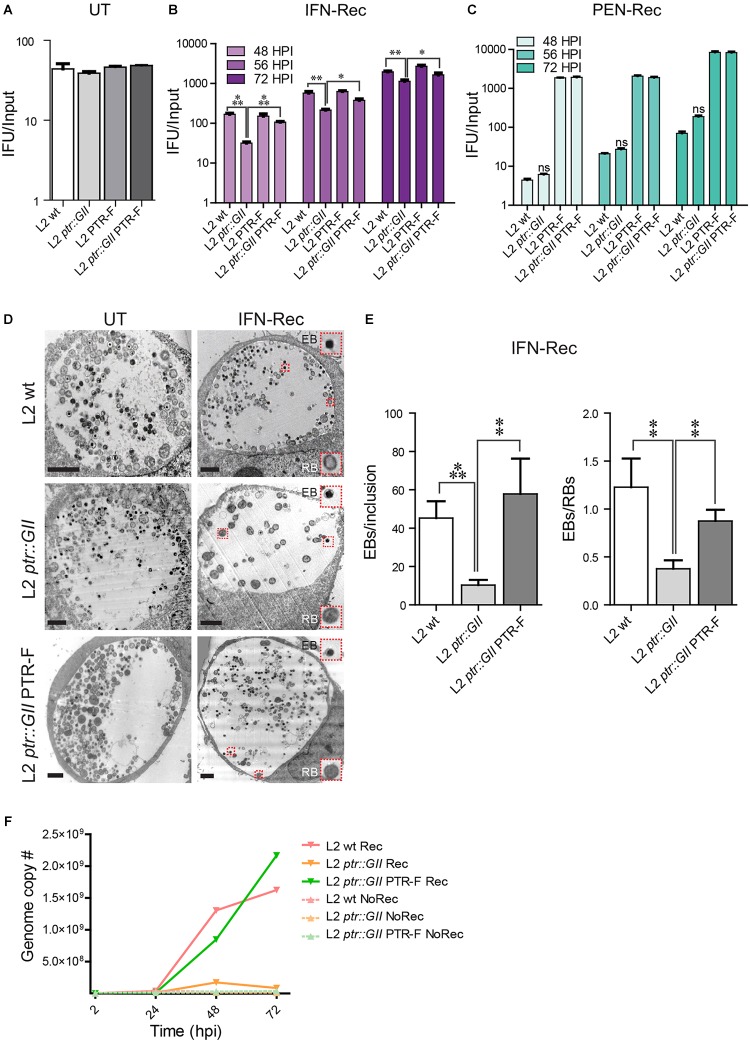
Insertional inactivation of *ptr* leads to impaired recovery of *C. trachomatis* from IFNγ-induced stress. **(A)** LGV-L2 *ptr* knock out (L2 *ptr::GII*), LGV-L2 transformed with the plasmid vector pBOMB-ptrF expressing FLAG-tagged Ptr (L2 PTR-F) and LGV-L2 *ptr* knock-out transformed with pBOMB-ptrF (L2 *ptr::GII* PTR-F), display similar levels of infectious progeny generation compared to *C. trachomatis* LGV-L2 wild type (L2 wt) strain in the untreated (UT) condition. The number of infectious bacteria was quantified at 30 hpi and normalized to the input (mean ± SEM, *n* = 3, Student’s *t*-test). **(B)** The *ptr* knock-out strain L2 *ptr::GII* exhibits decreased generation of infectious progeny upon recovery from IFNγ-induced stress (IFN-Rec), while this phenotype is rescued by expression of Ptr from a plasmid vector in the L2 *ptr::GII* PTR-F strain. HeLa cells were pre-treated with IFNγ (15 ng/mL) for 24 h and infected with the indicated strains. At 24 hpi, IFNγ was removed, cells were supplemented with tryptophan (100 mg/L) and incubated for various times post-infection to assess recovery. Infectious progeny post-recovery was quantified at 48, 56, and 72 hpi and normalized to the input (mean ± SEM). Statistical analysis was performed by Student’s *t*-test (*n* = 3; ^∗^*P* < 0.05, ^∗∗^*P* < 0.01, ^∗∗∗^*P* < 0.001). **(C)** L2 *ptr::GII* produces infectious progeny at similar levels than L2 wt upon recovery from penicillin-induced stress (PEN-Rec). HeLa cells were infected with the indicated strains and supplemented with penicillin (1 IU/mL) at the time of infection. At 24 hpi penicillin was removed, cells were replenished with complete medium and incubated up to 48, 56, or 72 hpi for recovery. Since pBOMB-ptrF carries ampicillin resistance, L2 PTR-F and L2 *ptr::GII* PTR-F are not affected by penicillin. In all cases, infectious progeny was normalized by input (mean ± SEM, *n* = 3). Statistical analysis was performed by Student’s *t*-test [non-significant (ns), *P* > 0.05]. **(D)** Representative TEM images of the untreated (UT, 30 hpi) and of IFN-Rec (48 hpi) conditions for HeLa cells infected the L2 wt, L2 *ptr::GII* and L2 *ptr::GII* PTR-F strains. Insets highlight *C. trachomatis* particles displaying typical ultrastructural features of EBs and RBs. Scale bars represent 2 μm. **(E)** Quantification of EBs/inclusion and EBs/RBs visualized by TEM during IFN-Rec. Values represent the mean ± SEM of at least three replicates. Statistical significance was calculated by Student’s *t*-test (^∗∗^*P* < 0.01 and ^∗∗∗^*P* < 0.001). **(F)** HeLa cells were treated with IFNγ for 24 h and infected with either L2 wt, L2 *ptr::GII* or L2 *ptr::GII* PTR-F strains. For the recovery condition (Rec), IFNγ was removed at 24 hpi and cells were replenished with full media supplemented with tryptophan. For the non-recovery condition (NoRec) IFNγ was continuously present during infection. Genome copy number was assessed by real-time PCR during IFNγ treatment and upon recovery at the indicated times post-infection using primers specific for *ompA*. Data points represent the mean of three replicates.

We next inactivated *ptr* in *C. trachomatis* LGV-L2 with a *ptr-*targeting group II intron (L2 *ptr::GII* strain) carrying a spectinomycin resistance cassette (Lowden et al., 2015). Disruption of the chromosomal copy of *ptr* in L2 *ptr::GII* was confirmed by PCR reactions and sequencing (Supplementary Figures S4, S5 and Supplementary Table S1). To complement Ptr expression, we transformed L2 *ptr::GII* with the plasmid pBOMB-ptrF, which expresses Ptr fused to a FLAG epitope (L2 *ptr::GII* PTR-F strain). As a control, we also transformed wild type *C. trachomatis* LGV-L2 with pBOMB-ptrF (L2 PTR-F strain) to determine if overexpression of Ptr from a plasmid elicits a detrimental effect. In the absence of stressors, all the strains generated similar levels of IFUs (Figure 2A) and exhibited similar growth rate in a one-step growth curve analysis (Supplementary Figure S6) compared to the wild type strain. This indicates that *ptr* is dispensable for the development of a productive cycle and that Ptr-FLAG overexpression is not detrimental to *C. trachomatis*. L2 *ptr::GII* was impaired for recovery from IFNγ- but not PEN-induced stress (Figure 2B,C), reproducing the phenotype observed for M275 (Supplementary Figure S2). Importantly, we observed that during the IFN-Rec condition, L2 *ptr::GII* transformed with the pBOMB-ptrF plasmid produced significantly more IFUs than L2 *ptr::GII* and similar levels of IFUs compared to L2 wt and L2-PTR-F control strains (Figure 2B), indicating that ectopic expression of Ptr rescued the ability to reactivate after IFNγ-induced stress in the *ptr* knockout strain. A similar assessment of recovery from penicillin-induced stress could not be made for strains transformed with pBOMB-ptrF, because this plasmid confers ampicillin resistance and thus the transformed strains were not affected by penicillin (Figure 2C).

An ultrastructural analysis by electron microscopy indicated that in the UT condition, L2 *ptr::GII* displayed no apparent alterations compared to L2 *ptr::GII* PTR-F and L2 wt (Figure 2D). However, during recovery from IFNγ-induced stress, L2 *ptr::GII* inclusions contained significantly less EBs and a reduced EB/RB ratio (Figure 2E), reproducing the phenotype of the original M275 mutant (Supplementary Figure S2E). Importantly, this decrease in total EB numbers and EB/RB ratio was rescued in the complemented strain (Figure 2D,E). Next, we determined if the reduction in IFU generation and EB/RB ratio observed in L2 *ptr::GII* was associated with defects in genome replication during recovery from IFNγ-induced stress. We assessed the rate of genome accumulation (Figure 2F) and found that in the presence of IFNγ, all strains showed a marked reduction in genome replication. Upon removal of IFNγ, L2 *ptr::GII* exhibited reduced genome accumulation compared to L2 wt. Importantly, the complemented strain L2 *ptr::GII* PTR-F restored the rates of genome accumulation to levels seen for the L2 wt control. Altogether, these findings provide genetic confirmation that loss of Ptr is associated with impaired recovery from IFNγ-induced stress.

**FIGURE 3:**
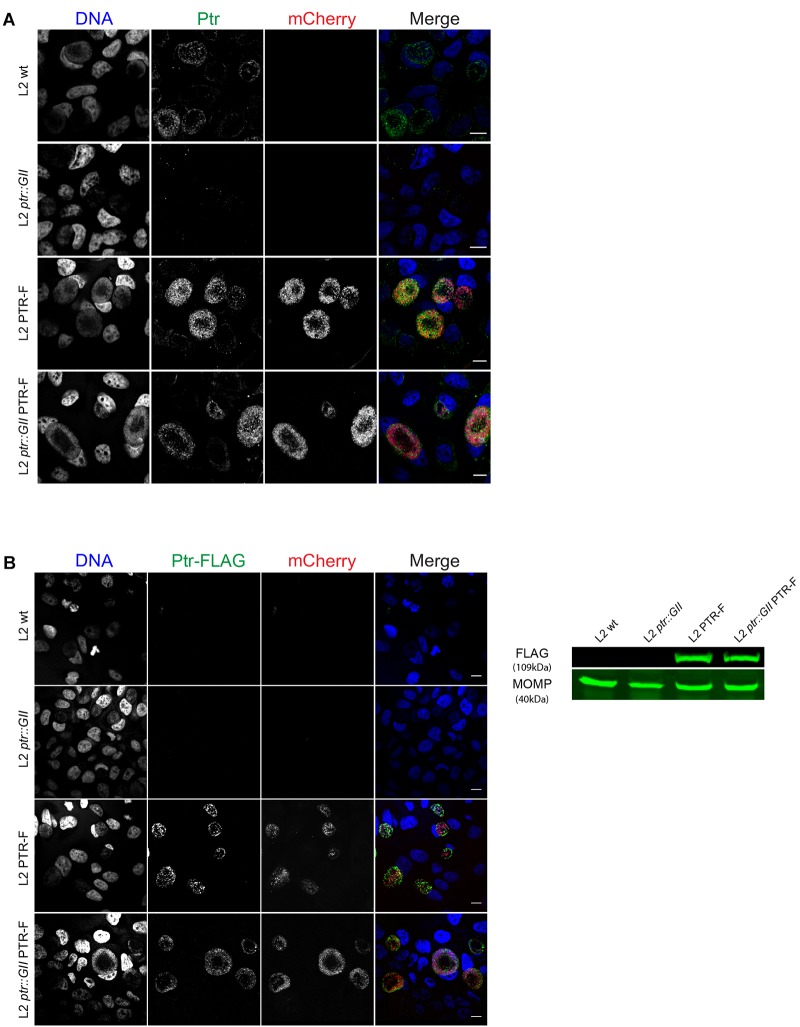
Insertional disruption of *ptr* results in lack of Ptr expression and strains transformed with pBOMB-ptrF express FLAG-tagged Ptr **(A)** Representative confocal images of HeLa cells infected with the indicated strains at 30 hpi. Ptr expression (green) was observed in wild type LGV-L2 (L2 wt, endogenous levels), LGV-L2 expressing FLAG-tagged Ptr (L2 PTR-F) and LGV-L2 *ptr* knock-out expressing FLAG-tagged Ptr (L2 *ptr::GII* PTR-F) strains but not in LGV-L2 *ptr* knock-out (L2 *ptr::GII*). Bacteria transformed with pBOMB-ptrF express mCherry (red). DNA was stained with Hoechst (blue). Scale bars, 10 μm. **(B)** Left panel: representative confocal images of HeLa cells infected with the indicated strains at 30 hpi. FLAG-tagged Ptr (green) and mCherry (red) highlight inclusions only in cells infected with L2 PTR-F and L2 *ptr::GII* PTR-F. DNA was stained with Hoechst (blue). Scale bars, 10 μm. Right panel: immunoblot with anti-FLAG M2 antibodies (FLAG) confirms expression of the expected ∼109 kDa FLAG-tagged Ptr in total protein extracts obtained from HeLa cells infected with L2 PTR-F or L2 *ptr::GII* PTR-F. For loading control, anti-MOMP antibodies were used (MOMP).

We further confirmed expression of Ptr in complemented strains by immunofluorescence microscopy with anti-Ptr antibodies. Ptr labeling was detected in the inclusion of L2 wt, L2 PTR-F and L2 *ptr::GII* PTR-F strains, but not in L2 *ptr::GII*, as expected (Figure 3A). We also verified that strains transformed with pBOMB-ptrF expressed FLAG-tagged Ptr both by immunofluorescence and immunoblots with anti-FLAG antibodies (Figure 3B).

### Ptr Is Expressed at Mid-to-Late Stages of *C. trachomatis* Developmental Cycle and Localizes to the Lumen of Inclusions

**FIGURE 4:**
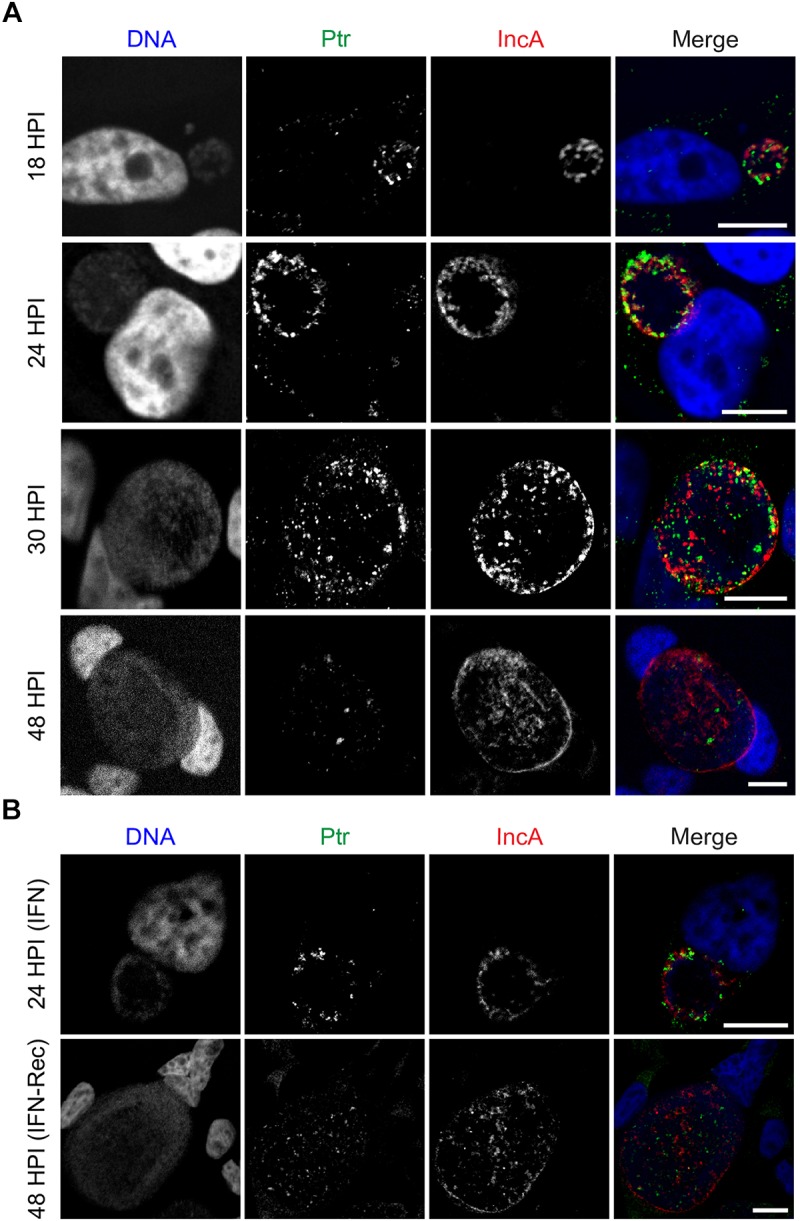
Ptr is expressed at mid to late stages of *C. trachomatis* developmental cycle. **(A)** Representative micrographs of HeLa cells infected with LGV-L2 at 18-24-30-48 hpi. **(B)** Representative images of HeLa cells infected with LGV-L2 during IFNγ treatment (IFN: cells were pre-treated for 24 h with 15 ng/mL of IFNγ, then infected for 24 h in presence of IFNγ) and upon recovery from IFNγ-induced stress (IFN-Rec: at 48 hpi, 24 h after removal of IFNγ and addition of tryptophan). Anti-Ptr (green) shows Ptr expression and anti-IncA (red) marks the boundaries of the inclusion membrane. Confocal images are depicted. Scale bars, 10 μm.

**FIGURE 5:**
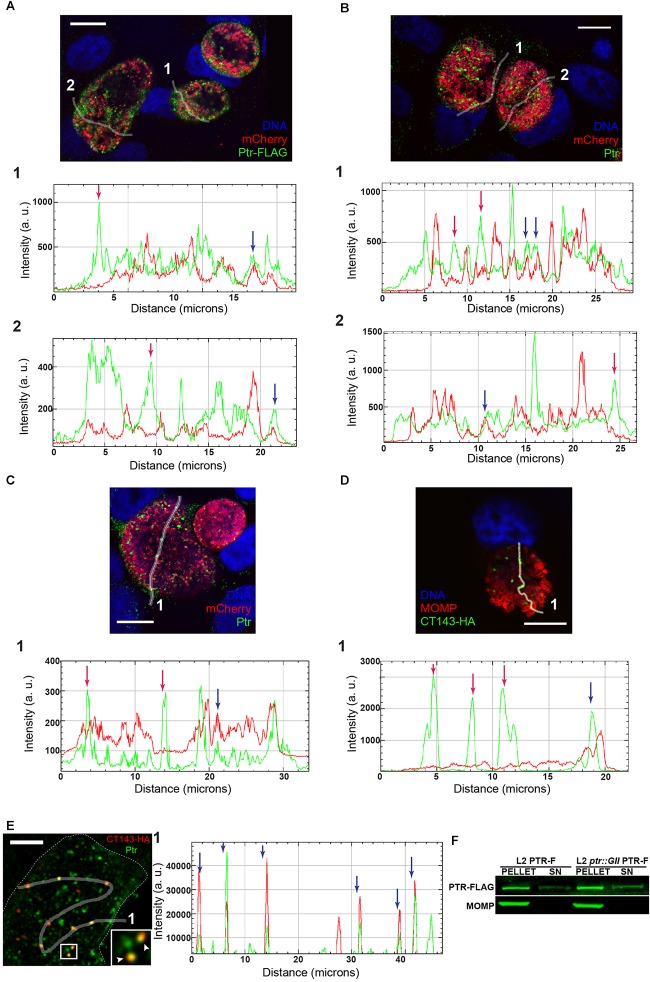
Ptr localizes to extra-bacterial particles within the lumen of the inclusion. **(A)** Upper panel: representative image of HeLa cells infected with LGV-L2 *ptr* knock-out expressing FLAG-tagged Ptr (L2 *ptr::GII* PTR-F) at 30 hpi. Freehand lines regions of interest crossing the inclusion (ROIs) 1 and 2 are indicated. Lower panels: multichannel plot profiles corresponding to the indicated ROIs (1, 2) depict the fluorescence intensities (a. u., arbitrary units) of Ptr-FLAG (green) and mCherry-expressing bacteria (red). **(B)** Upper panel: representative image of HeLa cells infected with LGV-L2 expressing FLAG-tagged Ptr (L2 PTR-F) at 30 hpi. ROIs 1 and 2 are indicated. Lower panels: multichannel plot profiles corresponding to the indicated ROIs (1, 2) depict the fluorescence intensities of FLAG-tagged and endogenous Ptr (green) and mCherry expressing bacteria (red). **(C)** Upper panel: representative image of HeLa cells infected with LGV-L2 expressing mCherry at 30 hpi. ROI 1 is indicated. Lower panel: multichannel plot profile corresponding to the indicated ROI (1) depict the fluorescence intensities of endogenous Ptr (green) and mCherry (red). Ptr was labeled with either anti-FLAG M2 **(A)** or anti-Ptr **(B,C)** antibodies. Bacteria constitutively express mCherry due to pBOMB-ptrF **(A,B)** or to p2TK2-SW2-mCherry **(C)** plasmids. **(A–C)** Note that FLAG-tagged Ptr and endogenous Ptr localize to bacteria (blue arrows) and to the intraluminal space within the inclusion (red arrows). **(D)** Upper panel: HeLa cells were infected with LGV-L2 expressing hemagglutinin (HA) epitope-tagged CT143, fixed at 30 hpi and labeled with anti-HA and anti-MOMP. CT143-HA localizes to extra-bacterial particles within the inclusion lumen (da Cunha et al., 2017). ROI 1 is indicated. Lower panel: multichannel plot profile corresponding to the indicated ROI (1) depict the fluorescence intensities of CT143-HA (green) and MOMP (red). Red arrows indicate CT143 localization outside the bacteria, blue arrow indicates CT143-HA labeling surrounded by anti-MOMP signal. DNA was stained with Hoechst. Confocal images are depicted. **(E)** Left panel: co-localization of Ptr and CT143-HA in HeLa cells at 30 hpi. A confocal single z-section is depicted. Inset highlights co-localizing structures (arrowheads). Right panel: multichannel plot profile corresponding to the indicated ROI in left panel depict the intensities of red (CT143-HA) and green (Ptr) fluorescence. Blue arrow indicates co-localizing structures. A surface render of this inclusion performed with Huygens Professional Software is included in the movie Supplementary File S1. **(F)** Distribution of Ptr as assessed by fractionation of HeLa cells infected with L2 PTR-F or L2 *ptr::GII* PTR-F at 30 hpi. Bacterial pellets (PELLET) and supernatants (SN) were immunoblotted with anti-FLAG (Ptr-FLAG) and anti-MOMP (MOMP). Scale bars represent 10 μm **(A–D)** and 5 μm **(E)**.

We assessed the expression of Ptr by immunofluorescence at different stages during the developmental cycle in HeLa cells infected with *C. trachomatis* LGV-L2. Endogenous Ptr expression was detected at 18, 24, and 30 hpi, and barely detected at 48 hpi, suggesting that at late stages of the developmental cycle the expression of this protein decreases (Figure 4A). In addition, we determined that Ptr is expressed during IFNγ-induced stress and recovery (Figure 4B). By immunofluorescence microscopy, HeLa cells infected with L2 *ptr::GII* PTR-F displayed anti-FLAG labeling localizing to bacteria and to the intraluminal space within inclusions (Figure 5A). Immunostaining of Ptr with anti-Ptr antibodies mirrored the localization pattern observed with anti-FLAG, both at ectopically expressed and endogenous levels (Figure 5B,C), emphasizing the specificity of the anti-Ptr antibodies. Overall, our immunostaining suggests that Ptr is present within inclusions and outside of the bacteria cells. Ptr contains a putative signal peptide for Sec-dependent secretion, indicating that this protein may be secreted into the inclusion lumen. It has been previously reported that other chlamydial proteases like HtrA, Tsp and CPAF are secreted and localize to organism-free vesicles within the lumen of inclusions (Zhong, 2011). In addition, other *Chlamydia* proteins like Pls1 and Pls2 (Jorgensen and Valdivia, 2008) and the type III secretion system substrates CT142, CT143, and CT144 (da Cunha et al., 2017) have also been reported to localize outside of the bacterial cells but within the inclusion lumen. To determine if Ptr co-localized with any of the previously characterized secreted intra-inclusion proteins, we transformed a plasmid expressing CT143 fused to an HA epitope tag (pCT143-2HA, da Cunha et al., 2017) into *C. trachomatis* LGV-L2 and assessed the subcellular localization of CT143 and Ptr by immunofluorescence microscopy. The localization of CT143 in *C. trachomatis* LGV-L2 transformed with pCT143-2HA reproduced the intraluminal localization pattern previously reported (da Cunha et al., 2017). Interestingly, CT143-2HA and Ptr partially co-localized with extra-bacterial particles within the inclusion (Figure 5E). A surface render of the inclusion shown in Figure 5E further supports the observation that Ptr can be found closely associated to CT143 positive structures in the inclusion lumen (movie Supplementary File S1).

To independently confirm secretion of Ptr, we carried out fractionation of HeLa cells infected with *C. trachomatis* LGV-L2 strains expressing Ptr-FLAG to separate bacterial cells (pellet) from host cell lysate (supernatants). As observed in Figure 5F, by immunoblot analysis the major *Chlamydia* outer membrane protein MOMP was only detected in the bacterial pellets, while Ptr-FLAG was detected both in the bacterial pellets and in the supernatants. Overall, these results lead us to postulate that a significant portion of Ptr is released from *Chlamydia* cells and localize to extra-bacterial particles within the inclusion lumen.

## Discussion

We used a collection of chemically mutagenized *C. trachomatis* strains (Kokes et al., 2015) to identify *C. trachomatis* genes potentially involved in recovery from IFNγ- and/or penicillin-induced stress. In this report, we characterized one mutant, M275, which showed impaired recovery from IFNγ- but not penicillin-induced stress. This mutant harbored 6 SNVs, one of them leading to a nonsense mutation in *ptr* gene. We disrupted *ptr* in *C. trachomatis* LGV-L2 with a type II intron (TargeTronR, Lowden et al., 2015) and generated a *ptr* null strain (L2 *ptr::GII*) that reproduced the defect observed for M275. Moreover, complementation of Ptr expression from a plasmid restored the generation of infectious progeny upon removal of IFNγ, confirming that *ptr* is required for recovery from IFNγ-induced stress in *C. trachomatis*. This gene encodes an uncharacterized secreted protease, which shares 23% identity with the zinc-dependent protease III of *E. coli* K-12 (Pitrilysin, a periplasmic enzyme that degrades small peptides). Ptr belongs to the M16 peptidase family and includes a conserved motif between amino acids 105–109 (H-X-X-E-H) in the N-terminal section involved in enzymatic activity in Pitrilysin. An InterPro database analysis of Ptr shows that the domain architecture of this protein is shared by other peptidases of related and distant species. Ptr is highly conserved across *C. trachomatis* serovars (>99% identity), while identity level with Ptr orthologs varies considerably across *Chlamydia* species (*C. muridarum* 82%, *C. pneumoniae* 47%, and *C. psittaci* 46%).

A previous screen for *C. trachomatis* mutants impaired for recovery upon removal of IFNγ used a GFP-expressing LGV-L2 library as a primary read-out for the number of inclusions formed in untreated compared to IFNγ recovery conditions (Muramatsu et al., 2016). In this screen, IFNγ-induced stress was achieved by pre-treating HeLa cells for 24 h with 10 ng/mL of IFNγ followed by infection for 24 h in presence of the stressor, while recovery was assessed 24 h after cells were washed and replenished with tryptophan-free media supplemented with indole. Our screen instead used a rifampin resistant LGV-L2 library of mutants and the read-out was based on quantifying infectious progeny generation upon recovery from the stressing condition. Another difference is that we used 15 ng/mL of IFNγ for stress induction and assessed recovery 24 h after cells were washed and replenished with media supplemented with an excess of tryptophan. Remarkably, in this independent screen Muramatsu et al. (2016) identified a defective mutant, Sip5, with amino acid substitutions in Ptr/CTL0175 (P831S) and in CTL0641 (H37Y). However, this mutant was not characterized further, so it remains unknown if the amino acid substitution in Ptr was involved in the defect observed for this strain.

Our results indicate that abrogation of Ptr expression causes reduced recovery from IFNγ-induced stress due to impaired RB to EB differentiation and reduced genome accumulation (Supplementary Figures S2D–F and Figure 2D–F). Muramatsu et al. (2016) also found mutants displaying altered rates of genome accumulation and EB generation upon removal of IFNγ. For instance, Sip6 (CTL0694^P105L^) presented uncoupled genome replication and EB production whereas Sip2 (CTL0225^G77E^) displayed decreased and delayed genome accumulation. These findings suggest that altered genome accumulation and RB to EB transition may be a cause of impaired recovery from IFNγ-induced stress.

The expression of *ptr* mRNAs in *C. trachomatis* LGV-L2 is relatively constant between 9 and 18 hpi in untreated condition and no significant changes were observed in iron-depleted conditions (Brinkworth et al., 2018). A transcriptional study focused on IFNγ-induced persistence and reactivation in *C. trachomatis* serovar D reported that *ptr* transcript levels were reduced ∼ 2 fold during IFNγ treatment and removal (Belland et al., 2003). Proteomics studies reported that Ptr is expressed in all *C. trachomatis* developmental forms (Saka et al., 2011; Ostergaard et al., 2016; Skipp et al., 2016). Saka et al. (2011) and Skipp et al. (2016) proteomic studies found that Ptr is expressed at higher levels in RBs compared to EBs, and scores among the top 30 most abundant proteins in *C. trachomatis* LGV-L2 RBs. A proteomic study by Ostergaard et al. (2016) was carried out in *C. trachomatis* serovar D and reported that Ptr levels did not change significantly in EBs, RBs or aRBs. Interestingly, this study found that under IFNγ-induced stress, the *C. trachomatis* serovar D proteome accumulates lower levels of tryptophan-rich proteins. Given that *C. trachomatis* Ptr has a relatively low tryptophan content compared to its *E. coli* homolog, it has been suggested that *C. trachomatis* Ptr has evolved to be expressed under IFNγ-induced tryptophan deprivation (Lo et al., 2012). In our study, we were not able to reliably measure Ptr expression levels by western-blot, since the antibody we generated only worked for immunofluorescence. However, our immunofluorescence observation that Ptr levels seemed higher at 24 hpi compared to 48 hpi is in agreement with previous proteomic data showing that Ptr is enriched in RBs compared to EBs in *C. trachomatis* LGV-L2 (Saka et al., 2011; Skipp et al., 2016).

Our immunolocalization and cell fractionation studies indicate that Ptr is released into the luminal space within the inclusion, where it can be found outside of bacteria cells (Figure 5). Interestingly, a subset of Ptr was found closely associated to the type III-secreted protein CT143, which has also been shown to localize inside the inclusion but outside of the *Chlamydia* organisms (da Cunha et al., 2017). This association, however, does not necessarily indicate that these two proteins interact and if there is a functional implication for this association, it remains to be determined.

## Conclusion

Our work provides evidence that Ptr, a poorly characterized protease, is secreted into the inclusion lumen and participates in *C. trachomatis* exit from IFNγ- but not penicillin-induced stress *in vitro*. The precise molecular mechanism by which Ptr mediates these effects remains to be determined. Since in human cells IFNγ-induced stress in *C. trachomatis* is mediated primarily by deprivation of an amino acid (tryptophan), it is tempting to speculate that the Ptr protease may be useful as an amino acids provider during recovery. This could explain why Ptr loss is associated to impaired recovery from IFNγ- but not penicillin-induced stress. Further studies with strains harboring mutations in the putative protease active site might be useful for uncovering the potential role of Ptr peptidase activity during IFNγ-induced stress and recovery.

## Ethics Statement

For antibody generation, mice were maintained in the SPF animal facilities with environmental enrichment that meet the conditions of the Guide to the Care and Use of Experimental Animals (CICUAL N° 001-2017).

## Author Contributions

MP, RV, and HS substantially contributed to the conception and design of the work. MP, HS, RB, AL, and MD acquired, analyzed, and interpreted the data. HS, MP, RV, RB, AL, and MD drafted and critically revised the manuscript. All authors approved the final version to be published and agree to be accountable for all aspects of the work.

## Conflict of Interest Statement

The authors declare that the research was conducted in the absence of any commercial or financial relationships that could be construed as a potential conflict of interest.

## Footnotes

1 http://tools.iedb.org/
