# Supplementary figures and images for: Ptr/CTL0175 Is Required for the Efficient Recovery of Chlamydia trachomatis From Stress Induced by Gamma-Interferon
Source: Front Microbiol. 2019 Apr 10;10:756. doi: 10.3389/fmicb.2019.00756 (PMC6467971; doi:10.3389/fmicb.2019.00756)

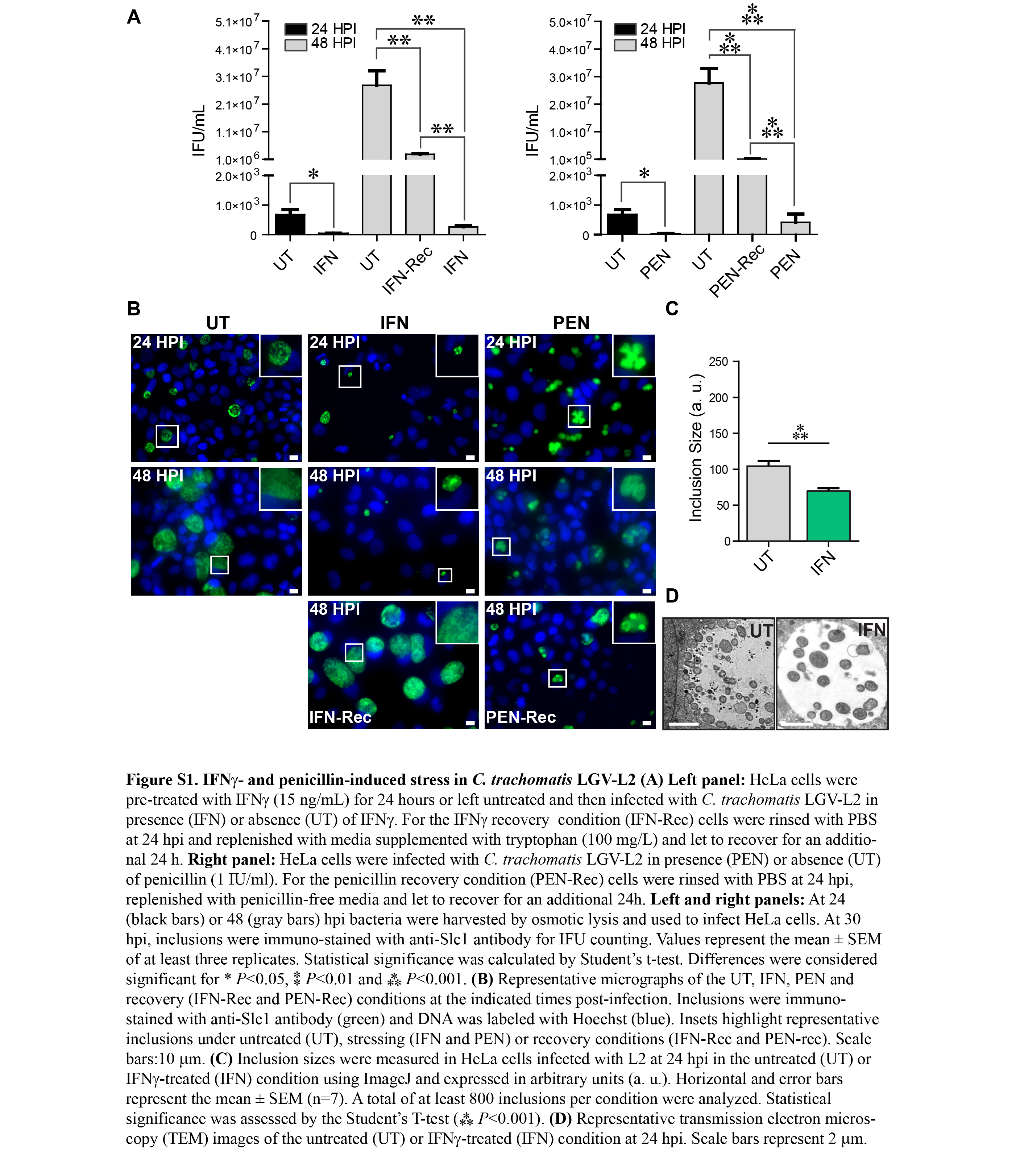

Supplement: Supplementary file 1 [file Image_1.jpg]

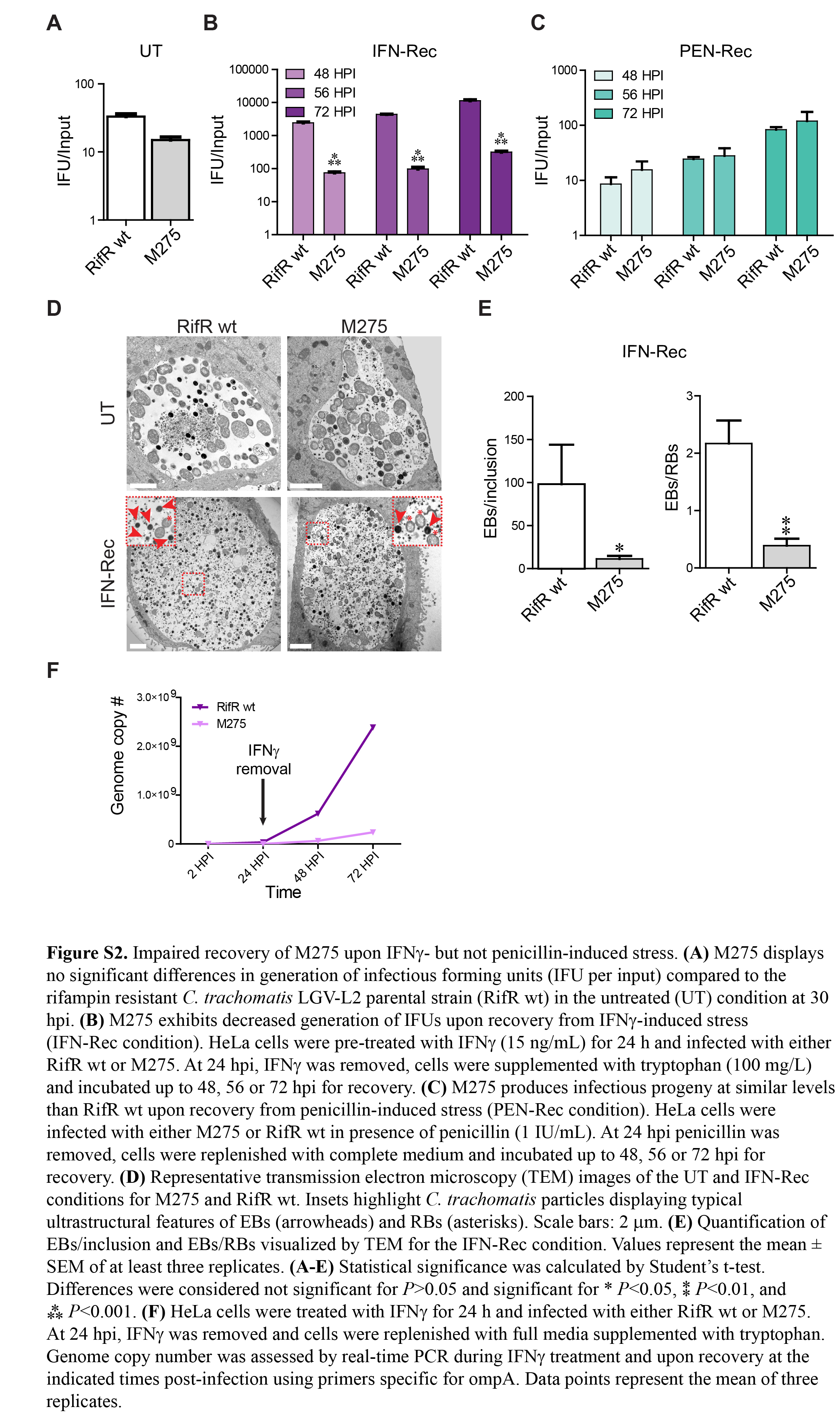

Supplement: Supplementary file 2 [file Image_2.JPEG]

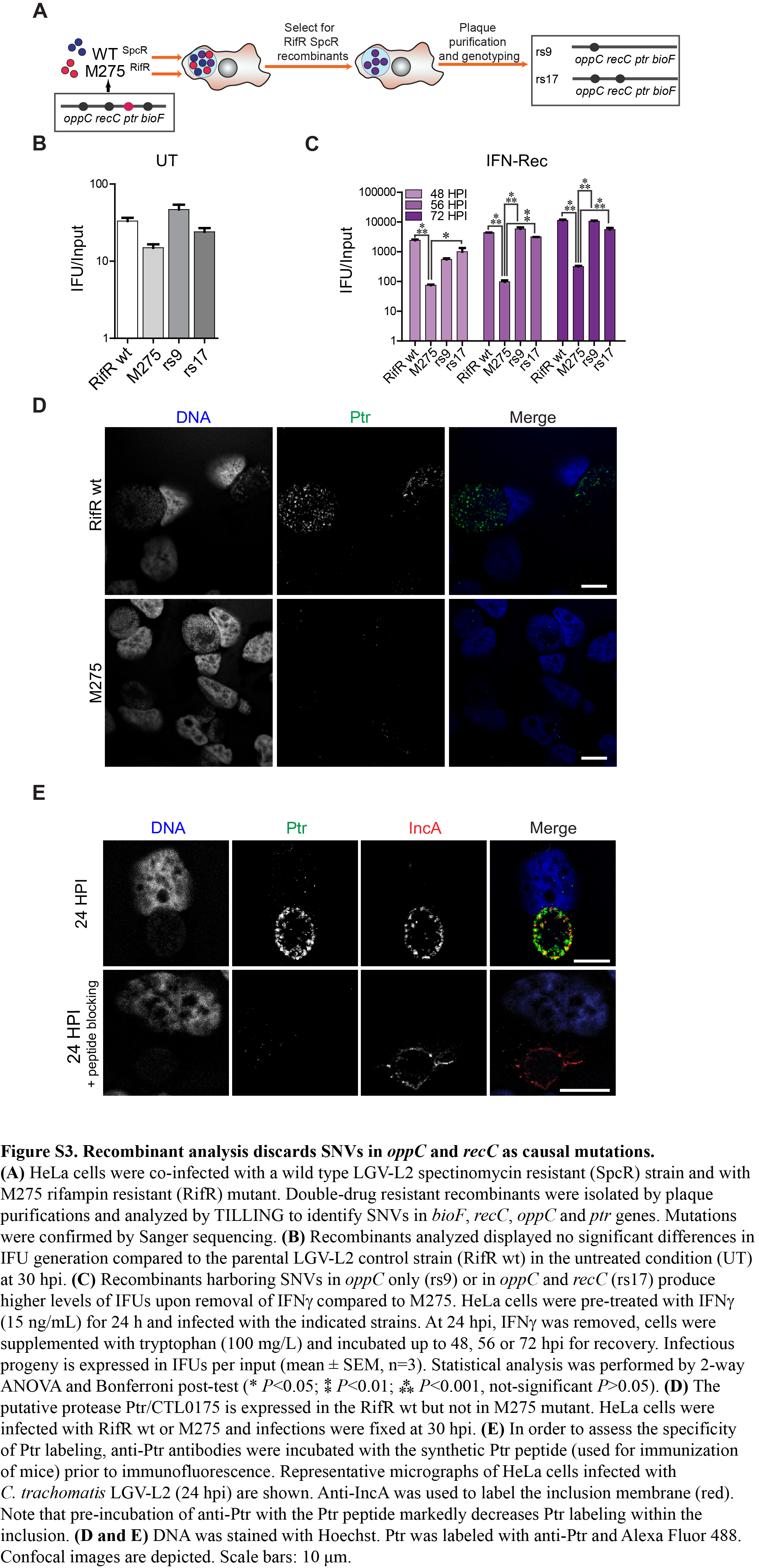

Supplement: Supplementary file 3 [file Image_3.jpg]

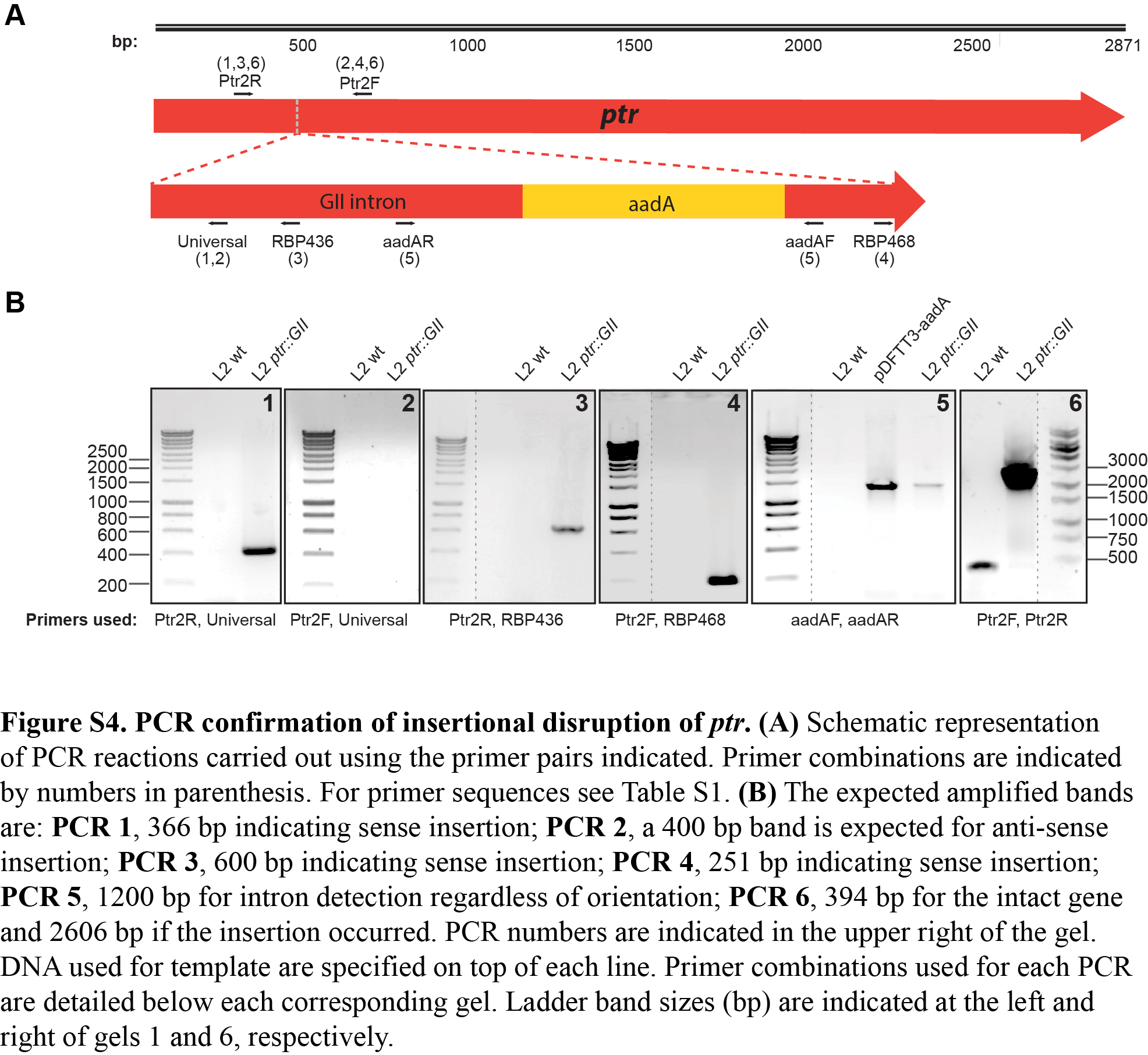

Supplement: Supplementary file 4 [file Image_4.JPEG]

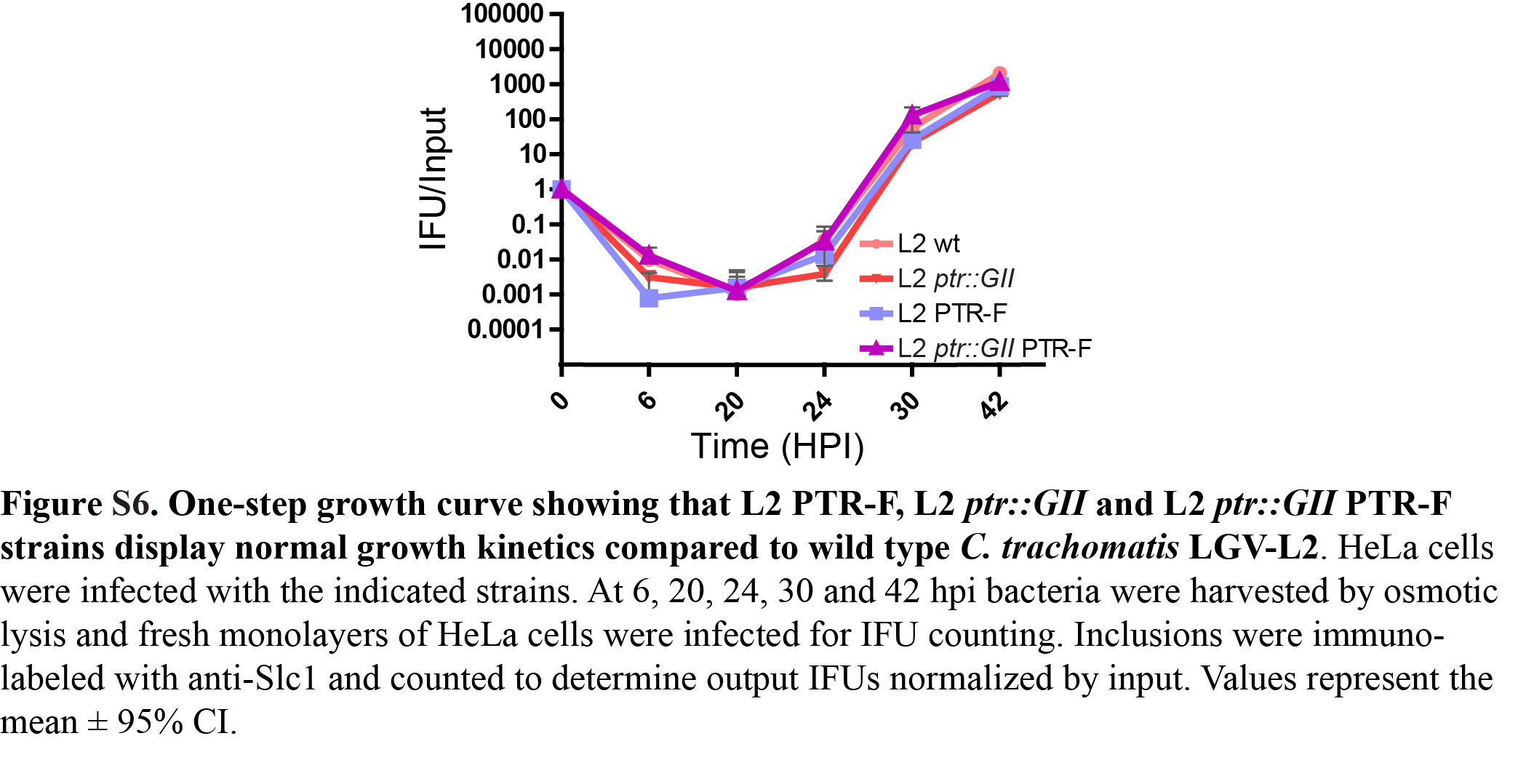

Supplement: Supplementary file 6 [file Image_6.jpeg]
