# Supplementary material for: Ptr/CTL0175 Is Required for the Efficient Recovery of Chlamydia trachomatis From Stress Induced by Gamma-Interferon
Source: Front Microbiol. 2019 Apr 10;10:756. doi: 10.3389/fmicb.2019.00756 (PMC6467971; doi:10.3389/fmicb.2019.00756)
Supplement: Supplementary file 5 [file Data_Sheet_1.PDF]

A

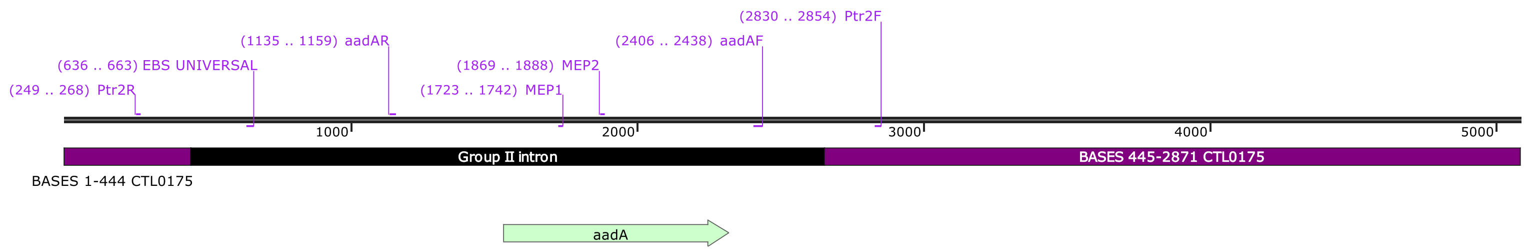

B

CGGAGCCGCTTTAGTTGTCAAAACAGGAAATAATGCAGATCCTGTCGAATTCCCAGGCTTAGCCCATTTTACAGAACACTGTGTGTTCCTCGGGAATGAAAAATATCCCGAGCCCTCAG
GATTTCCTGCCTTCCTAAGCACACATGGGGGTATCTATAACGCATTTACTTACCCAGATAAAACCTGCTTCCTATTCGTGCGCCCAGATAGGGTGTTAAGTCAAGTAGTTTAAGGTACT
ACTCTGTAAGATAACACAGAAAACAGCCAACCTAACCGAAAAGCGAAAGCTGATACGGGAACAGAGCACGGTTGGAAAGCGATGAGTTACCTAAAGACAATCGGGTACGACTGAGTCGC
AATGTTAATCAGATATAAGGTATAAGTTGTGTTTACTGAACGCAAGTTTCTAATTTCGGTTAAGCATCGATAGAGGAAAGTGTCTGAAACCTCTAGTACAAAGAAAGGTAAGTTAGAGA
ATAGGACTTATCTGTTATCACCACATTTGTACAATCTGTAGGAGAACCTATGGGAACGAAACGAAAGCGATGCCGAGAATCTGAATTTACCAAGACTTAACACTAACTGGGGATACCCT
AAACAAGAATGCCTAATAGAAAGGAGGAAAAAGGCTATAGCACTAGAGCTTGAAAATCTTGCAAGGGTACGGAGTACTCGTAGTAGTCTGAGAAGGGTAACGCCCTTTACATGGCAAAG
GGGTACAGTTATTGTGTACTAAAATTAAAAATTGATTAGGGAGGAAAACCTCAAAATGAAACCAACAATGGCAATTTTAGAAAGAATCAGTAAAAATTCACAAGAAAATATAGACGAAG
TTTTTACAAGACTTTATCGTTATCTTTTACGTCCAGATATTTATTACGTGGCGACGCGTTGCCTGACGATGCGTGGAGACCGAAACCTTGCGCTCGTTCGCCAGCCAGGACAGAAATGC
CTCGACTTCGCTGCTGCCCAAGGTTGCCGGGTGACGCACACCGTGGAAACGGATGAAGGCACGAACCCAGTGGACATAAGCCTGTTCGGTTCGTAAGCTGTAATGCAAGTAGCGTATGC
GCTCACGCAACTGGTCCAGAACCTTGACCGAACGCAGCGGTGGTAACGGCGCAGTGGCGGTTTTCATGGCTTGTTATGACTGTTTTTTTGGGGTACAGTCTATGCCTCGGGCATCCAAG
CAGCAAGCGCGTTACGCCGTGGGTCGATGTTTGATGTTATGGAGCAGCAACGATGTTACGCAGCAGGGCAGTCGCCCTAAAACAAAGTTAAACATCATGAGGGAAGCGGTGATCGCCGA
AGTATCGACTCAACTATCAGAGGTAGTTGGCGTCATCGAGCGCCATCTCGAACCGACGTTGCTGGCCGTACATTTGTACGGCTCCGCAGTGGATGGCGGCCTGAAGCCACACAGTGATA
TTGATTTGCTGGTTACGGTGACCGTAAGGCTTGATGAAACAACGCGGCGAGCTTTGATCAACGACCTTTTGGAAACTTCGGCTTCCCCTGGAGAGAGCGAGATTCTCCGCGCTGTAGAA
GTCACCATTGTTGTGCACGACGACATCATTCCGTGGCGTTATCCAGCTAAGCGCGAACTGCAATTTGGAGAATGGCAGCGCAATGACATTCTTGCAGGTATCTTCGAGCCAGCCACGAT
CGACATTGATCTGGCTATCTTGCTGACAAAAGCAAGAGAACATAGCGTTGCCTTGGTAGGTCCAGCGGCGGAGGAACTCTTTGATCCGGTTCCTGAACAGGATCTATTTGAGGCGCTAA
ATGAAACCTTAACGCTATGGAACTCGCCGCCCGACTGGGCTGGCGATGAGCGAAATGTAGTGCTTACGTTGTCCCGCATTTGGTACAGCGCAGTAACCGGCAAAATCGCGCCGAAGGAT
GTCGCTGCCGACTGGGCAATGGAGCGCCTGCCGGCCCAGTATCAGCCCGTCATACTTGAAGCTAGACAGGCTTATCTTGGACAAGAAGAAGATCGCTTGGCCTCGCGCGCAGATCAGTT
GGAAGAATTTGTCCACTACGTGAAAGGCGAGATCACCAAGGTAGTCGGCAAATAATGTCTAACAATTCGTTCAAGCCGACGCCGCTTCGCGGCGCGGCTTAACTCAAGCGTTAGATGCA
CTAAGCACATAATTGCTCACAGCCAAACTATCAGGCCCGGGACGCGTTGGGAAATGGCAATGATAGCGAAACAACGTAAAACTCTTGTTGTATGCTTTCATTGTCATCGTCACGTGATT
CATAAACACAAGTGAATGTCGACAGTGAATTTTTACGAACGAACAATAACAGAGCCGTATACTCCGAGAGGGGTACGTACGGTTCCCGAAGAGGGTGGTGCAAACCAGTCACAGTAATG
TGAACAAGGCGGTACCTCCCTACTTCACTCCGTAAACAATGCAGATTTAGATAATGCTCTGGATCAGTTTGTACATCTCTTTATCCAACCTCTCTTCCGCCAAGAAGATTTAAACAAAG
AAGTTCATGCTGTTGAGCAAGAATTTGCGATGCATCCAACTAAAGATTCTCGTCGTATGCATCGTATTCAACAACTTATAGCTCCTAAAAATCACCCATTAAAACGC

Figure S5. Confirmation of intron insertion site (A) Schematic representation of intron insertion in *ctl0175* showing primers used for sequencing. **(B)** Sequence of the intron insertion site in LGV-L2 *ptr* knock-out (L2 *ptr::GII*) strain. Primers used are highlighted: Ptr2F, black; Ptr2R, yellow; aadA_R, pink; aadA_F, dark gray; EBS Universal, green; MEP1, light blue; MEP2, red. Spectinomycin resistance cassette and *ptr* sequences are indicated in red and blue letters, respectively. Group II intron sequence is indicated in black.
