# Supplementary material for: Ptr/CTL0175 Is Required for the Efficient Recovery of Chlamydia trachomatis From Stress Induced by Gamma-Interferon
Source: Front Microbiol. 2019 Apr 10;10:756. doi: 10.3389/fmicb.2019.00756 (PMC6467971; doi:10.3389/fmicb.2019.00756)
Supplement: Supplementary file 7 [file Data_Sheet_2.PDF]

Table S1. Primers used in this study.

Primer	Sequence 5' to 3'
Ptr_2_EBS2	TGAACGCAAGTTTCTAATTTCGGTTAAGCATCGATAGAGGAAAGTGTCT
Ptr_2_ EBS1/delta	CAGATTGTACAAATGTGGTGATAACAGATAAGTCCTATTCTCTAACT TACCTTTCTTTGT
Ptr_2_IBS1/2	AAAAAAGCTTATAATTATCCTTATGCTTCCTATTCGTGCGCCCAGATAGGG TG
Universal	CGAAATTAGAAACTTGCGTTCAGTAAAC
recC_gene_F	CGAAGAACTGTTTCAACCCTACG
recC gene_R	AGGAACGACAACTTCCCATGT
bioF_gene_F	AGTTTCCGCCATAATGATCTGGA
bioF_gene_R	GAGGACAAGCGACTCCAACA
oppC_gene_F	TCCCGAACATGCTCTTCAGT
oppC_gene_R	TGGTAAGCTAAAGAATGAATGCCT
Ptr_gene_F	TCTAACTTTGCAGAAGCTTCGT
Ptr_gene_R	TCACAGTACTTTTTGAAATAGCTGT
Ptr2F	GCGTTTTAATGGGTGATTTTTAGGA
Ptr2R	CGGAGCCGCTTTAGTTGTCA
aadaF	GTAACGCGTCCCGGGCCTGATAGTTTGGCTGTGAG
aadaR	TCTACGCGTTGCCTGACGATGCGTGGAG
RBP436	AGTGCTATAGCCTTTTTCCTCCT
RBP468	TGGTGCAAACCAGTCACAGT
MOMP_F	TTTGCCGCTTTGAGTTCTGC
MOMP_R	CGCCGAAACCTTCCCATAGA
F-rpoBPtr	GCCCGCGGACATGGACAACCACCTTCCTGTTATCA
R-PtrFLAG-R	CGGGTACCCTACTTGTCATCGTCATCCTTGTAATCGATGTCATGATCTTTA TAATCACCGTCATGGTCTTTGTAGTCTTTTTCCTGCGACGAGTTGGCT
FompA_BamHI	ATATTTGGATCCCGATGAAAAAACTCTTGAAATCG
RompA_SalI	ATTGAGTCGACGAAGCGGAATTGTGCA
